# Therapeutic potential of *Aloe vera* in diabetes mellitus treatment: an update

**DOI:** 10.1007/s44446-026-00070-6

**Published:** 2026-03-04

**Authors:** Muhammad Adil, Sumayya Akram, Matloob Ahmad, Magdi E. A. Zaki

**Affiliations:** 1 Department of Chemistry, Government College University Faisalabad, Faisalabad, 38000 Pakistan; 2 Department of Chemistry, College of Science, Imam Mohammad Ibn Saud Islamic University (IMSIU), 11623 Riyadh, Saudi Arabia

**Keywords:** *Aloe vera*, Diabetes mellitus, *α*-glucosidase, *α*-amylase, Inhibitor

## Abstract

Diabetes mellitus is a metabolic disease manifested by hyperglycemia, leading to critical health challenges in the kidneys, eyes, nerves, and cardiovascular system. *Aloe vera* (*Aloe barbadensis miller*), a traditionally used herbal plant for treating various diseases, contains abundant bioactive compounds like alkaloids, flavonoids, acemannans, anthraquinones, chromones, anthrones, enzymes, vitamins, and minerals. These compounds are responsible for its diverse biological activities, including antimicrobial, anticancer, antidiabetic, anti-inflammatory, antioxidant, and hypolipidemic etc. This review primarily focuses on diabetes treatment through enzyme inhibition and modulation of metabolic parameters, with additional emphasis on diabetic complications including, the wound healing efficacy of *Aloe vera*. This study aims to evaluate the potential of *Aloe vera* in managing diabetes and minimizing the chances of diabetes-related organ damage. It was done by inhibition of several enzymes, including *α*-glucosidase, *α*-amylase, dipeptidyl peptidase-4, pancreatic lipase, sucrase, and maltase enzymes, both *in vivo* and *in vitro.* The previous research revealed that administrating various doses of *Aloe vera*-based extracts via orally and non-orally routes in streptozotocin-induced diabetes offered metabolic regulation of signaling pathways like blood sugar, insulin, lipid profile, body weight, oxidative stress, TNF-*α*, and IL-6 levels. Moreover, clinical data have confirmed *Aloe vera*’s potential in diabetic wound healing by promoting tissue regeneration mechanisms like angiogenesis, fibrinogenesis, collagen synthesis, and inflammation to reduce lesion size. These therapeutic properties highlight the hypoglycemic nature of *Aloe vera*, promising its potential for future clinical use as an alternative for diabetes treatment.

**Graphical Abstract:**

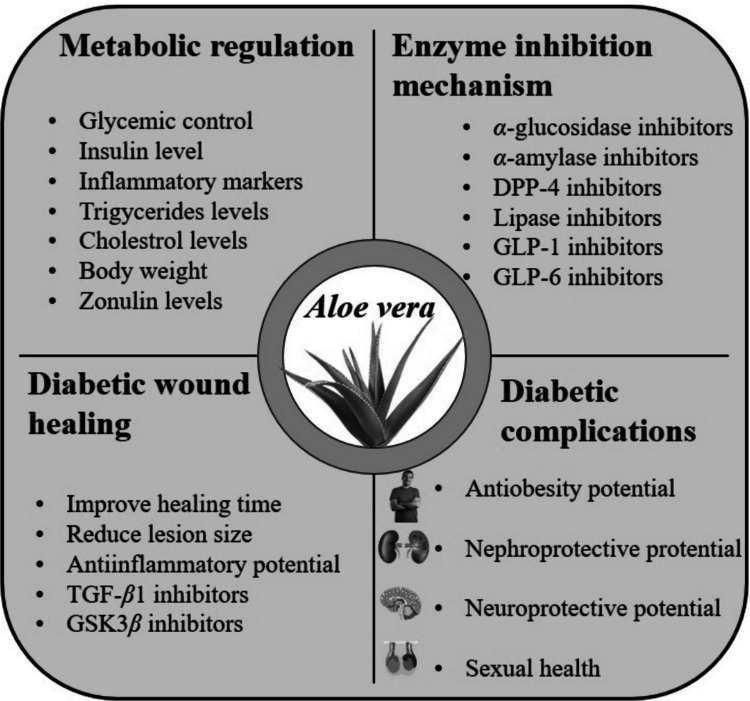

## Introduction

Diabetes mellitus is common and estimated to be the world’s fastest-growing metabolic disease, associated with chronic hyperglycemia, resulting from defects in insulin secretion, and action, or both (Bilous et al. 2021; Hill-Briggs et al. 2020). Hyperglycemia may exhibit symptoms like polyuria, polyphagia, and polydipsia, while prolonged unmanaged hyperglycemia leads to various complications including cardiomyopathy, angiopathy, nephropathy, and retinopathy (Kazeem et al. 2020; Eckstein et al. 2017). According to the American Diabetes Association (ADA), diabetes mellitus is a persistent and intricate condition that mandates lifetime healthcare to mitigate severe complications (Association AD 2020).

According to the International Diabetes Federation (IDF), approximately 589 million adults are diabetics, with 11.1% prevalence worldwide, in which Pakistan is reported to have a 34.5 million adult populations in 2024; however, the number is estimated to rise to 853 million diabetics worldwide and 70.2 million in Pakistan overtaking the USA in 2050. According to 2024 report, more than 3.4 million people (9.3% of global death) are died due to diabetes (Ceriello and Colagiuri 2025).

There are three types of diabetes mellitus (DM), i.e., type-1 DM, type-2 DM, and gestational DM (Antar et al. 2023). Type 1 diabetes mellitus, also named insulin-dependent or juvenile diabetes, is a chronic autoimmune disease mainly present in children and adults caused by a deficiency of enough insulin due to the destruction of pancreatic *β*-cells, ultimately resulting in hyperglycemia (Katsarou et al. 2017; Oliveira et al. 2023). Type 2 diabetes is the most common non-insulin-dependent type, which occurs due to insulin secretory dysfunction or insulin resistance of cells which leads to glycemic imbalance (Galicia-Garcia et al. 2020). Third type is gestational diabetes, which often appears during pregnancy due to hormonal change resulting in glucose resistance, while mostly it disappears after childbirth (Zhu and Zhang 2016; Berberoglu 2019).

**Fig. 1:**
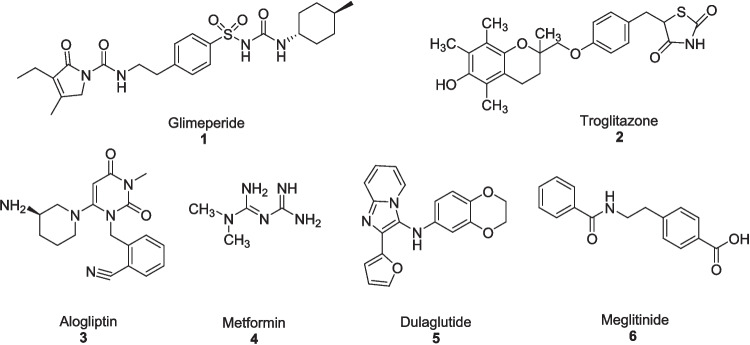
Anti-diabetic medications

The metabolic changes in diabetes contributed to excessive mitochondrial superoxide production in endothelial cells of vessels and myocardium. This oxidative imbalance caused diabetic complications, including both microvascular (involving eyes, kidneys, and nerves) and macrovascular (involving heart defects) complications (Giacco and Brownlee 2010). In early ages, diabetes was medicated by only controlled diet to live longer (Mazur 2011), while first effective drug was insulin, discovered in 1921 (Lewis and Brubaker 2021). The treatment of diabetes has become a worldwide challenge (Vaz and Patnaik 2012). To overcome these challenges, different heterocyclic compounds are used as drugs as they exhibited diverse biological properties like antioxidant (Aslam et al. 2014), antibiotic (Ahmad et al. 2012), antiviral (Ahmed et al. 2014; Khalid et al. 2015) and antidiabetic (Akram et al. 2024; Ibraheem et al. 2020), antioxidant (Sahar et al. 2017) as well as monoamine oxidase inhibitor (Ahmad et al. 2018; Abid et al. 2017; Zaib et al. 2015). Hence, different classes of heterocyclic based medications have demonstrated antidiabetic activity including, glimperide (sulfonylurea) **1** (Scheen 2021), troglitazone (thiazolidinediones) **2** (Day 1999) and alogliptins (gliptins) **3** (Fisman and Tenenbaum 2015) are widely used. Others classes of oral medications like metformin (biguanides) **4** (Magno et al. 2022; Raczyńska et al. 2018), dulaglutide (glucagon-like peptide agonists) **5** (Collins and Castello 2024; Willard et al. 2012) and meglitinides **6** (Guardado-Mendoza et al. 2013; Levien et al. 2001) are also utilized (Fig. 1).

The vast spectrum of various options disrupts the prioritization of drug therapy; in addition, these medications have serious side effects like liver dysfunction, obesity, and cardiac disorders (Grant et al. 2007; Hippisley-Cox and Coupland 2016; Sumida et al. 2020; Provilus et al. 2011). While prolonged use of antidiabetic chemical drugs can affect kidneys and enhance cancer risks by 1.36 times (But et al. 2014). To counter these side effects, there is a need to explore alternative therapies and the potential of herbal plants, which have fewer adverse effects (Fallah et al. 2012; Pandey et al. 2011). Herbal plants have been used for many years by human beings to prevent and manage diseases; moreover, almost 25% of drugs are derived from plants (Choudhury et al. 2018; Radha and Laxmipriya 2015).

**Fig. 2:**
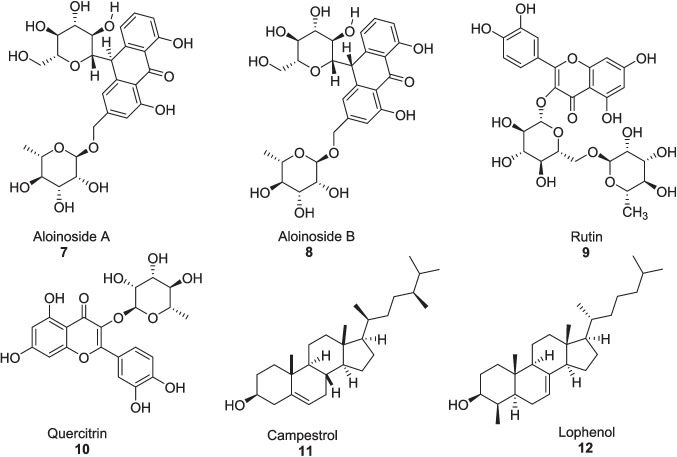
Anti-diabetic agents from *Aloe vera*

*Aloe vera* is a miracle plant having a wide range of biological activities like anti-diabetic (Muñiz-Ramirez et al. 2020), anti-inflammatory (Devaraj and Karpagam 2011), anticancer (Shalabi et al. 2015), antihyperlipidemic (Fallah et al. 2012), antioxidant (Nejatzadeh-Barandozi 2013), antibacterial (Jain et al. 2016), antifungal (Danish et al. 2020), antiulcer (Widyastiwi and Oktaviani 2018), dermatological (Sharma et al. 2014), wound healing (Oryan et al. 2019), and gastroprotective activities (Maan et al. 2018; Park et al. 2017). *Aloe vera* has been used traditionally as an antihyperglycemic agent for a longer time, making it a potential aid in the management of diabetes and pre-diabetes (Ezuruike and Prieto 2014; Kumar et al. 2019). *Aloe vera* contains many bioactive compounds like aloinoside A **7**, aloinoside B **8**, rutin **9**, quercitrin **10**, campesterol **11** and lophenol **12** which behave as antidiabetic agent (Fig. 2) (Sharma et al. 2021). *Aloe vera* is a medicinal plant having a therapeutic effect on blood glucose and insulin sensitivity (Budiastutik et al. 2022). The earliest use of *Aloe vera *via topical administration was documented in 1935 (Collins and Collins 1935), and its antidiabetic effect was first reported in 1985 by Agarwal (Agarwal 1985). *Aloe vera* had been proved an effective supplement with standard antidiabetic medications like metformin, sulfonylurea and glibenclamide (Bunyapraphatsara et al. 1996; Shoaib et al. 2022; Desai et al. 2024).

Previous literature reviews had information gap about enzyme inhibition mechanism of *Aloe vera* and its beneficial impact in diabetic complication including chronic wound healing aspects that needs to be further elucidated (Hutapea and Susanto 2021; Saleem et al. 2021). Over past years, many studies about *Aloe vera* in diabetes management and its wound-healing potential were reported (Hekmatpou et al. 2019; Shakib et al. 2019; Sánchez et al. 2020; Sharma et al. 2021; Haghani et al. 2022). The present study focused on the studies from 2020–2025 in order to ensure novelty and highlight the most recent research and clinical studies. This study provides an up-to-date overview of *Aloe vera* as a natural antidiabetic agent, with comprehensive explanation of enzymatic inhibition mechanisms, formulation evaluations (serum, gel, juice, and extracts), diabetic related complications and wound healing potential. This comprehensive review critically covers recent studies from 2020 to 2025, highlighting the potential of *Aloe vera* as a natural therapeutic agent for diabetes management and its health related issues.

## Methodology

Literature for comprehensive review was retrieved from Google Scholar based on keywords: *Aloe vera* in diabetes management, Antidiabetic activity of *Aloe vera*, *Aloe vera* and enzyme inhibition, *Aloe vera* in diabetic complications, *Aloe vera* in diabetic wound healing from 2020–2025. A total of 233 articles were retrieved, of which 136 met the inclusion criteria and were added in this review.

## Anti-diabetic activity of Aloe Vera

### Enzyme inhibition mechanism

Govindarajan et al. (2021) investigated streptozotocin (STZ) induced male diabetic rats in both in vitro and in vivo trials suggested that the *Aloe vera* carbohydrate fraction (AVCF) showed a potential anti-diabetic effect by activating sugar uptake, and inhibiting* α*-amylase and *α*-glucosidase. AVCF was isolated by the aqueous extraction of its gel leading to ethanol precipitation and rotary evaporation techniques. The findings indicated that AVCF inhibited *α*-amylase and *α*-glucosidase with IC_50_ values of 60.44 ± 1.02 μg and 82.85 ± 1.05 μg, respectively in a dose-dependent manner against standard drug Acarbose at *p *< 0.0001. In vitro studies, 20 and 60 μg/ml dosages of AVCF in RIN-m5F rat cells elevated the Brmodeoxyuridine (BrdU) and insulin levels by 58.2 ± 4.1%, 67.5 ± 1.6% and 40.2 ± 0.2%, 58.5 ± 0.3%, respectively. Moreover, the inflammatory mediators including TNF-*α* was diminished by 66.5 ± 1.5%, 72.7 ± 1.5% and that of IL-6 decreased by 65.3 ± 1.2% and 71.81 ± 1.5%, respectively. The in vivo studies, 48 STZ induced (40 mg/kg BW) diabetic male wistar rats of weight 180–200 g were treated with two doses of AVCF for 3 weeks. The in vivo administration with the low dose of 27 mg/kg BW was ineffective whereas high dosage of 54 mg/kg BW could elevate insulin level by 73.08 ± 1.5% and declined the glucagon, fasting plasma of glucose, TNF-*α*, IL-6 and sPLA2 levels by 51.26 ± 1.2% and 74.8 ± 3.40%, 57.4 ± 0.9%, 56.1 ± 1.2%, and 71.7 ± 0.1%, respectively. Additionally, potent dose of AVCF boosted hexokinase and glycogen synthase enzyme by 53.9 ± 0.4% and 50.6 ± 1.0%, leading to an increase in the glycogen level by 87.9 ± 1.5% but inhibiting the glucose-6-phosphatase by 50.6 ± 1.0% (*p *< 0.0001) in diabetic rats.

**Fig. 3:**
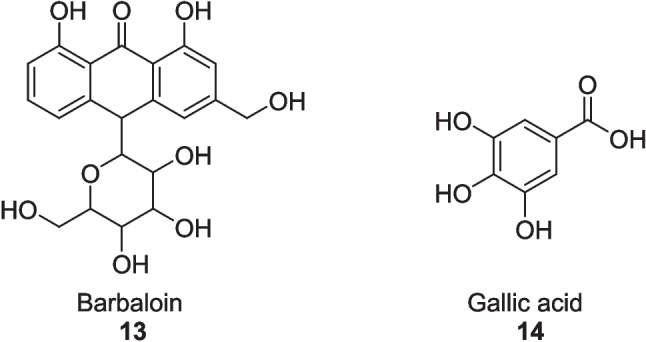
Compounds extracted from ethyl acetate fraction of *Aloe vera*

The ethyl acetate fraction of *Aloe vera* and its extracted ingredients were evaluated by Ghosh et al. (2025) as an antidiabetic agent in both in vivo and in vitro studies, offering *α*-glucosidase, *α*-amylase, and *α*-lipase inhibition. In vitro study, the ethyl acetate fraction of *Aloe vera* exhibited strong inhibition of *α*-glucosidase with an IC_50_ value of 164.48 ± 1.15 μg/ml, *α*-amylase with an IC_50_ value of 223.41 ± 3.28 μg/ml, and *α*-lipase with an IC_50_ value of 290.29 ± 2.24 μg/ml in comparison with the chloroform fraction having IC_50_ values of 244.63 ± 3.65 μg/ml, 312.15 ± 4.65 μg/ml, and 1009.8 ± 3.42 μg/ml, respectively. While the extracted compounds barbaloin **13** and gallic acid **14** (Fig. 3) inhibited *α*-glucosidase with IC_50_ values of 169.91 ± 4.24 μg/ml and 220.58 ± 3.82 μg/ml, *α*-amylase with IC_50_ values of 228.54 ± 3.71 μg/ml, and *α*-lipase with IC_50_ values of 275.02 ± 2.58 μg/ml and 390.80 ± 3.32 μg/ml against the Acarbose having IC_50_ values of 146.69 ± 0.78 μg/ml, 240 ± 3.83 μg/ml, and 271.21 ± 3.67 μg/ml, respectively. In vivo study, all treatments of *Aloe vera* significantly elevated the insulin level and alleviated the fasting blood sugar and glycated hemoglobin (HbA1c) in STZ-induced diabetic rats at *p* < 0.01 after 28 days. It also significantly reduced the total cholesterol, triglyceride levels, and low-density lipoprotein (LDL) level, whereas it boosted the high-density lipoprotein (HDL) level at *p* < 0.01 to facilitate the lipid profile metabolism. Oxidative stress was also attenuated due to enhanced glutathione and catalase activities, while decreasing glutamate oxaloacetate transaminase (GOT), glutamate pyruvate transaminase (GPT), and alkaline phosphatase (ALP) enzymes improved hepatic function at *p* < 0.01 in a dose-dependent approach.

**Fig. 4:**
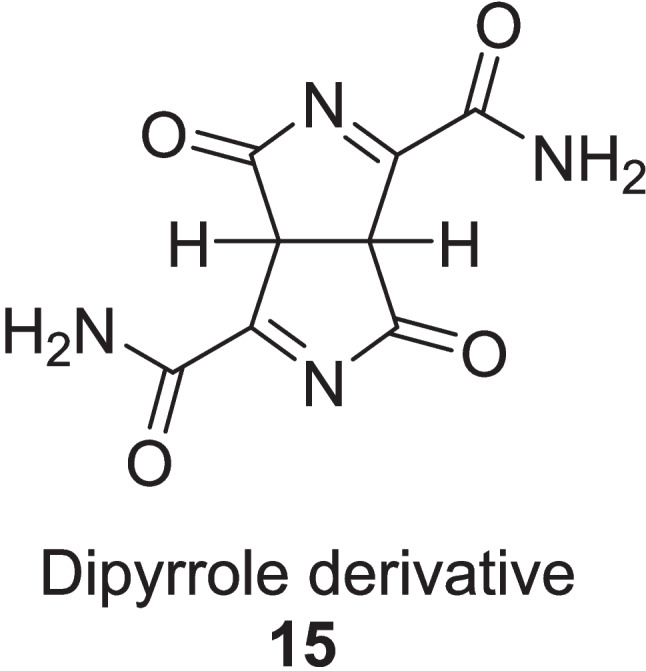
Dipyrrole derivative as DPP-IV inhibitor

Prasannaraja et al. (2020) extracted dipyrrole derivative **15**, from *Aloe vera* gel by gel filtration that acted as dipeptidyl peptidase-IV (DPP-IV) enzyme inhibitors to control the diabetes (Fig. 4). The in vitro studies were performed on the blood plasma of diabetic rats and the extracted compound was found to be potent non-competitive DPP- IV enzyme inhibitor having IC_50_ value of 8.59 ± 2.61 µM and Ki of 4.7 ± 0.038 µM.

The polypeptide fraction of the *Aloe vera* gel was evaluated by Babu et al. (2021a, b) as an alternative treatment for diabetes type-1 by lowering the intestinal permeability and zonulin concentration via glucagon-like peptide-1 (GLP-1)/DDP-IV routes in 42 STZ-induced diabetic male Wistar rats for 21 days. The PPF was extracted by ethanolic reflux extraction method. It was observed through *in vitro* studies that PPF (peptide/polypeptide fraction) acted as DPP-IV inhibitor with an IC_50_ value of 174.4 ± 2.3 μg/mL in a concentration-dependent fashion in RIN-m5F cells. PPF with doses of 10 μg/mL and 20 μg/mL were delivered, and a significant increase in insulin levels was observed by 55.1% and 68.2%, respectively, against the standard drug (Sitagliptin) by 67.2% with (*p* < 0.0001). Respective doses also effectively diminished the TNF-*α* levels by 56.8% and 59.5%, IL-6 levels by 54.9% and 61.6%, respectively, against the standard drug (Sitagliptin) as 57.1% and 63.9%, respectively. Two different doses of PPF were selected for *in vivo* studies, a dosage of 0.225 mg/kg exhibited negligible change, but highest dose of 0.450 mg/kg elevated insulin and zonulin levels to 145 ± 3.1 pmol/L and 4.9 ± 1.3 ng/mL, respectively, and glucagon levels were alleviated to 64.6 ± 1.2 pmol/L (*p* < 0.001).

**Fig. 5:**
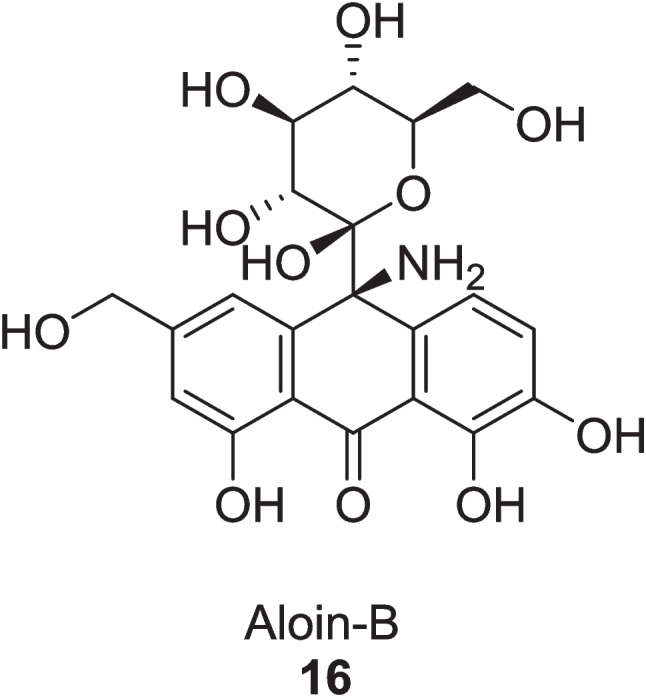
Aloin-B derivative as *α*-amylase inhibitor

Cahyaningtias and Sanjaya (2023) investigated the effect of *Aloe vera* extract, Aloin-B derivatives on type 2 diabetes mellitus by inhibiting the *α*-amylase enzyme by using LC–MS and QSAR techniques. The extract was isolated by ethanolic maceration with rotary-based drying techniques. Among all the extracted derivatives, the compound **16** was identified as most potent *α*-amylase inhibitor with a lowest binding energy of −7.07 kcal/mol and an inhibition constant of 6.58 µM (Fig. 5). It also followed Lipinski rule and has LD_50_ value of 2.509 kcal/mol. The compound **16** could also be used as a potent oral drug, as it satisfied the authentication standards with correlation coefficient of 0.980 and PRESS value of 0.0004.

Muñiz-Ramirez et al. (2020) reported the beneficial effects of methanolic extract of *Aloe vera* leaves as anti-diabetic agent by inhibiting the* α*-amylase, *α*-glucosidase, pancreatic lipase enzymes and the advanced glycation protein reaction. The *Aloe vera* methanolic extract (AVM) potent dose of 5 mg/ml demonstrated significant suppression of *α*-amylase, *α*-glucosidase and pancreatic lipase enzymes by 87%, 66% and 15% against Acarbose as standard control. *In vitro* study, the same dose significantly decreased fructosamine value from 119.6 mM to 77.5 mM against a standard aminoguanidine solution of 75.8 mM. The AVM optimal dose also inhibited the glycation reaction of bovine serum albumin (BSA), with inhibition rates evaluated via bovine albumin glycosylation, methylglyoxal-treated BSA, arginine-treated methylglyoxal and N*ε*-carboxymethyl lysine (CML) were 85.64%, 65%, 65% and 73%, respectively against the standard reference aminoguanidine solution within same timeframe.

**Fig. 6:**
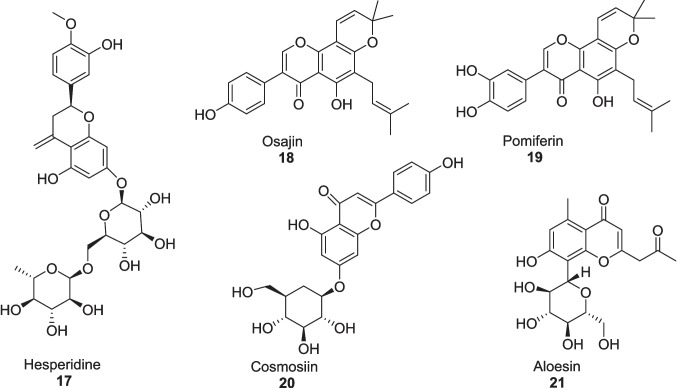
Bioactive compounds of *Aloe vera* as *α*-amylase and *α*-glucosidase inhibitors

Zakaria et al. (2023) conducted in silico analysis of five active compounds from *Aloe vera* that acted as *α*-amylase and *α*-glucosidase inhibitors to reduce the blood glucose levels in diabetes mellitus. Among the five different compounds, the respective compounds hesperidine **17**, osajin **18**, pomiferin **19** and cosmosiin **20** exhibited *α*-glucosidase inhibition with the binding efficiencies of −8.8 kcal/mol, −7.8 kcal/mol, −8.1 kcal/mol, −7.7 kcal/mol, respectively much lower than standard acarbose as −7.3 kcal/mol (Fig. 6). Compound **17** also exhibited as *α*-amylase inhibitor with a binding efficiency of −9.1 kcal/mol against acarbose, −8.5 kcal/mol. While compound aloesin **21** did not exhibit strong inhibition for both enzymes. The pharmacokinetic and toxicity prediction showed that these bioactive compounds were found to be more potent as their absorption rate in the gastrointestinal pathway was in between 31.48–96.59%. Additionally, these compounds showed no toxicity and have no potential to hepatoxicity according to AMES testing.

Govindarajan and Ayesha (2023) reported the effect of the anti-diabetic potential of *Aloe vera* carbohydrate fractions via diethylaminoethyl cellulose on pancreatic *β*-cell in STZ-triggered Rin-m5F and HepG2 cells, and the sugar composition was determined through chromatographic techniques. The extract was extracted via aqueous extraction and ethanol precipitation techniques. Among ten fractions, six fractions were found to be *α*-amylase and *α*-glucosidase inhibitors, but only three fractions were found to be most effective. All these fractions contained different concentrations of *N*-acetyl neuraminic acid, melibiose, fructose, mannose, glucosamine, rhamnose, arabinose, fucose, ribose, mannose, sucrose, ribulose, psicose and glucosamine. Fraction **1** acted as *α*-amylase inhibitor with IC_50_ value of 14.2 ± 1.4 μg while fractions **5** and** 6** acted as *α*-glucosidase inhibitor with IC_50_ values of 19.06 ± 1.2 μg and 9.13 ± 0.5 μg, respectively. *In vitro* study, two different doses of fraction **1** (2 μg/mL and 6 μg/mL) significantly elevated the cell vitality, BrdU and insulin levels by 62.3% and 70.2%, 61.5 ± 3.6% and 71.6 ± 1.8% and 20.3 ± 1.4% and 34.2 ± 1.5%, respectively at *p* < 0.0001 in streptozotocin (STZ) induced (1 µM dose) Rin-m5F cells. Respective doses also alleviated the TNF-*α* by 58.1 ± 1.2% and 74.5 ± 2.1% and IL-6 levels by 70.2 ± 1.5% and 75.02 ± 1.4%, respectively at *p* < 0.0001. However, the lower dose (2 µM) of fraction **4** and higher dose (6 µM) of fraction **1** g activated the AMP-activated protein kinase enzyme (AMPK) by 8.46% and 33.4% (*p* < 0.0001) against standard Metformin by 4.8% (*p* < 0.01) in HepG2 liver cells in response to high glucose. The same doses of fraction **9** effectively deactivated the AMPK enzyme by 52.7% and 60.3% against standard reference, metformin by 49.3% and 56.7% at *p* < 0.0001, respectively, in response to low glucose level.

**Fig. 7:**
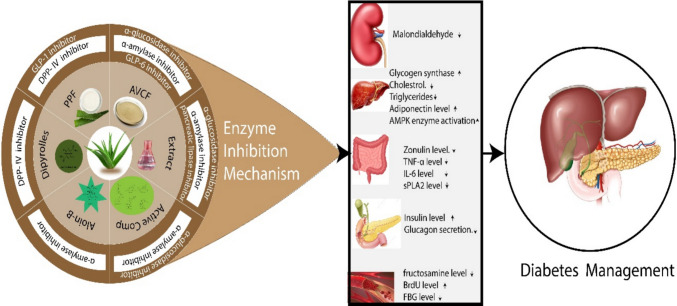
Enzyme inhibition mechanism of *Aloe vera*

*Aloe vera* extracts and its ingredients played a key role in diabetes management by inhibition of various enzymes like *α*-glucosidase, *α*-amylase, DPP-4, GLP-1, GLP-6 and pancreatic lipase enzymes. These respective enzymes restored the bio physiological parameters including blood glucose level, insulin level, triglycerides levels, cholesterol levels, inflammatory markers, BrdU and zonulin levels, which ultimately facilitated the diabetes management (Fig. 7). *Aloe vera* had no adverse effects *in vitro*, *in vivo* and in silico study and all studies were limited for a short-period of time (Zakaria et al. 2023; Govindarajan et al. 2021). Table 1 depicts the enzyme inhibition along with primary outcomes of *Aloe vera* based fractions and ingredients.

**Table 1:** Summary of Enzyme inhibition studies of *Aloe vera* in Diabetes treatment

Extract	Extraction Methods	Study type	Sample size	Dosages	Duration	Enzyme Inhibition	Results	Ref
AVCF	By aqueous extraction of *Aloe vera* gel, leaded with ethanol precipitation	In vitro	RIN-m5F cells	20 μg/ml	21 days	*α*-amylase IC_50_: 60.44 ± 1.02 μg *α*-glucosidase IC_50_: 82.85 ± 1.05 μg Glucose-6-phosphatase: IC_50_: 50.6 ± 1.0%	Increase: BrdU level: 58.2 ± 4.1% Insulin level: 40.2 ± 0.2% Decrease Inflammatory markers: TNF-* α*: 66.5 ± 1.5%; IL-6: 65.3 ± 1.2%	Govindarajan et al. (2021)
				60 μg/ml			Increase: BrdU level: 67.5 ± 1.6% Insulin level: 58.5 ± 0.3% Decrease Inflammatory markers: TNF-* α*: 72.7 ± 1.5%; IL-6: 71.81 ± 1.5%	
		In vivo	STZ-induced diabetic Male Wistar rats (180–200 g)	27 mg/kg	21 days		Ineffective	
				54 mg/kg			Increase: Insulin level: 73.08 ± 1.5% Glycogen level: 87.9 ± 1.5% Decrease: Glucagon level: 51.26 ± 1.2%; Fasting plasma of glucose:74.8 ± 3.40%; Inflammatory markers: TNF-* α*: 57.4 ± 0.9%; IL-6:56.1 ± 1.2% sPLA2:71.7 ± 0.1%; Boosting of Hexokinase: 53.9 ± 0.4% Glycogen synthase enzyme: 50.6 ± 1.0%	
Ethyl acetate fraction Barbaloin **13** Gallic acid **14**	Gel was collected from washed leaves, dried at 60 °C, grounded with petroleum ether and then extracted by ethanol (90%) via Soxhlet method	In vitro	Enzyme inhibition assays	Ethlyl acetate fraction 200 µg/mL; Barbaloin 15 mg/kg; Gallic acid: 15 mg/kg		Ethyl acetate Fraction: *α*-glucosidase IC_50_:164.48 ± 1.15 μg/ml *α*-amylase IC_50_:223.41 ± 3.28 μg/ml *α*-lipase IC_50_:290.29 ± 2.24 μg/ml **Barbaloin:** *α*-glucosidase IC_50_:169.91 ± 4.24 μg/ml *α*-amylase IC_50_:228.54 ± 3.71 μg/ml *α*-lipase IC_50_: 275.02 ± 2.58 μg/ml **Gallic acid:** *α*-glucosidase IC_50_: 220.58 ± 3.82 μg/ml *α*-amylase IC_50_: 440.81 ± 4.02 μg/ml *α*-lipase IC_50_:390.80 ± 3.32 μg/ml		Ghosh et al. (2025)
		In vivo	120 STZ-induced diabetic male Wistar rats		28 days		Lowered the total cholesterol, Triglycerides level, fasting blood sugar, HbA1c at *p* < 0.01	
*Aloe vera* gel	Extracted by gel filtration	In vitro	Blood plasma of diabetic rat	––	––	DPP-4 enzyme IC_50_:8.59 ± 2.61 µM	Ki: 4.7 ± 0.038 µM	Prasannaraja et al. (2020)
PPF of *Aloe vera* gel	By ethanolic reflux extraction	In vitro	RIN-m5F cells	10 μg/mL		DPP-4 enzyme IC_50_:174.4 ± 2.3 μg/mL	Increase Insulin level: 55.1% Decrease Inflammatory markers: TNF-*α*: 56.8%; IL-6: 54.9%	Babu et al. (2021a, b)
				20 μg/mL			Increase Insulin level: 68.2% Decrease Inflammatory markers: TNF-*α*: 59.5%; IL-6: 61.6%	
		In vivo	42 STZ-induced diabetic male Wistar rats	0.225 mg/kg	21 days		Negligible effect	
				0.450 mg/kg	21 days		Increase: Insulin levels: 145 ± 3.1 pmol/L; Zonulin levels: 4.9 ± 1.3 ng/mL; Decrease Glucagon level: 64.6 ± 1.2 pmol/L at *P* < 0.001	
Aloin-B derivatives	Isolated by ethanolic maceration followed by rotary drying techniques	In silico	––	––	––	*α*-amylase: Inhibition Constant: 6.58 µM Binding energy: −7.07 kcal/mol		Cahyaningtias and Sanjaya (2023)
Methanolic extract of *Aloe vera*	By washing, grinding of whole leaves then extracted 300 g of powder with 3 L of methanol via Soxhlet method	In vitro	Enzyme inhibition system, Glycation models	5 mg/ml	4 weeks	*α*-amylase: 87% *α*-glucosidase: 66% pancreatic lipase: 15%	Decrease Fructosamine value: 119.6 mM to 77.5 mM Inhibition of glycation reaction by: bovine albumin glycosylation: 85.64% methylglyoxal-treated BSA: 65% arginine-treated methylglyoxal: 65% N*ε*-CML: 73%	Muñiz-Ramirez et al. (2020)
Active compound from *Aloe vera* Comp **17, 18, 19, 20**	No physical extraction	In silico	Molecular docking			Binding efficiencies of; *α*-glucosidase: Comp **17**: −8.8 kcal/mol Comp **18**: −7.8 kcal/mol Comp **19**: −8.1 kcal/mol Comp **20**: −7.7 kcal/mol *α*-amylase: Comp **17**: −9.1 kcal/mol		Zakaria et al. (2023)
AVCF	Isolated by the aqueous extractein and then precipitated by ethanol, dried and stores	In vitro	RIN-m5F and HepG2 Cells	2 μg/mL and 6 μg/mL (Fraction **1**)	48 h	*α*-glucosidase: IC_50_: 19.06 ± 1.2 μg (Fraction **5**) IC_50_: 9.13 ± 0.5 μg (Fraction **6**) *α*-amylase: IC_50_: 14.2 ± 1.4 μg (Fraction **1**)	Increase: Cell vitality: 62.3% and 70.2% BrdU: 61.5 ± 3.6% and 71.6 ± 1.8% Insulin level:20.3 ± 1.4%and34.2 ± 1.5% Decrease Inflammatory markers: TNF-*α*: 58.1 ± 1.2% and 74.5 ± 2.1% IL-6: 70.2 ± 1.5% and 75.02 ± 1.4%, respectively at *p* < 0.0001	Govindarajan and Ayesha (2023)
				2 & 6 µM Fraction **4**			AMPK enzyme activation: 8.46% and 33.4%, respectively	
				2 & 6 µM Fraction **9**			AMPK enzyme deactivation: 52.7% and 60.3%, respectively	

As the enzymatic inhibition revealed the initial biochemical pathway by which *Aloe vera* and its active ingredients managed the hyperglycemia by acting on carbohydrate-metabolizing enzymes, including *α*-glucosidase and *α*-amylase, but these factors are not enough to explain its entire antidiabetic efficacy. The *in vivo* metabolic adjustments identified in rats or humans indicate that inhibition of enzymes laid the foundation for metabolic regulations, ultimately affecting the glycemic control, lipid metabolism, and inflammatory and oxidative mediators. Therefore, moving toward metabolic regulation revealed the effect of enzyme action at the systemic level, offering a mechanistic linkage between the enzyme inhibition mechanism and metabolic regulation.

### Metabolic regulation

Ramadhini and Ritonga (2022) showed the hypoglycemic effect of *Aloe vera* juice among 30 STZ-induced female white *Rattus norvegicus* rats treated with a dose of 1.8 ml/200 g BW orally for 12 days. At the start of the study, the blood glucose level of the positive group of mice was evaluated to be 498.60 ± 89.77 mg/dl. Whereas, administration of *Aloe vera* juice robustly mitigated the blood glucose level to 77.4 ± 8.89 mg/dl at *p* < 0.05 which were analyzed via Gluco-DR. At the same time, the live weight of mice was also elevated up to 167.4 ± 13.37 g, as it was dropped in diabetic rats from 201.90 ± 10.66 g (negative group) to 150.30 ± 10.41 g (positive group) at *p* < 0.05.

Fatima et al. (2024) showed the anti-hyperglycemic effect of *Aloe vera* serum (Dried leaf powder) on 25 STZ-induced diabetic male sprague–dawley rats treated with doses of 1.5 g, 3.0 g and 4.5 g for 24 days. *Aloe vera* leaves were washed, dried and then grinded to make powder. Hyperglycemic control was facilitated by lowering the thyroid hormones *i.e.,* triiodothyronine was significantly decreased at *p*-value of 0.12 (highly significant) while thyroxine declined insignificantly at *p* > 0.05. *Aloe vera* serum also exhibited anti-stress potential by lowering the cortisol levels at p-value of 0.00 (highly significant).

Javaid and Waheed (2020a) conducted a research to show the *Aloe vera’s* hypoglycemic activity on 40 STZ-induced type 2 diabetes sprauge-dawley rats by giving an oral dose of *Aloe vera* leaf extract (300 mg/kg) for 60 days *Aloe vera* extract was extracted via physical slicing and ambient drying leading to pulverization in the absence of solvent extraction. *Aloe vera* extract notably suppressed the mean fasting blood sugar value of STZ-induced diabetic rats from 498.40 mg/dl to 89.30 mg/dl against Sitagliptin (93.00 mg/dl), used as a standard drug at *p* < 0.001. It also effectively lowered the HbA1c from 11.84% to 4.02% against Sitagliptin (3.73%) at *p* < 0.001.

Javaid and Waheed (2020b) in another research reported that *Aloe vera* could also effectively lower the triglycerides level from 221.00 mg/dl to 112.50 mg/dl against Sitagliptin (125.00 mg/dl) at *p* < 0.001.

Hasan and Abdullah (2022) studied the antidiabetic effect of processed *Aloe vera* gel in 30 STZ-diabetic female albino rats administered with a dose of 300 mg/kg orally for 21 days. *Aloe vera* gel was extracted by the crude gel isolation method and then sterilized by 0.4 µm Millipore membrane. *Aloe vera* gel decreased the blood glucose, cholesterol and triglycerides levels markedly from 250 mg/dL, 97 mg/dL and 113 mg/dL to 122 mg/dL, 71 mg/dL and 102 mg/dL, respectively against the standard drug Glibenclamide at *p* ≤ 0.05.

The effect of *Aloe vera* leaf gel was investigated by Fazlani et al. (2020) towards blood glucose level and body weight in 24 alloxan monohydrate induced diabetic male albino rats treated orally at a dose of 1000 mg/kg BW for 21 days. *Aloe vera* gel was isolated from latex and rind mechanically and then sterilized by Ultraviolet rays. *Aloe vera* gel significantly alleviated the blood glucose level from 254.64 ± 1.50 mg/dL to 93.80 ± 0.60 mg/dL against standard Metformin 133.69 ± 0.74 mg/dl, while elevated the live body weight from 118.88 ± 0.60 g to 161.10 ± 0.74 g against Metformin 135.93 ± 1.26 g at *p* < 0.05.

Al-Sowayan and AL-Sallali (2023) reported that treatment with *Aloe vera* extracted aloin **13** was administered to 26 STZ-induced albino rats of weight 200–250 g at a dosage of 30 mg/kg for 30 days. Aloin was purchased commercially which was extracted from outer bark of *Aloe vera*. It empirically reduced the blood glucose sugar and boosted the insulin levels at *p* < 0.05. It also decreased the oxidative stress by lowering malondialdehyde (MDA) levels and raising the Congeners Glutathione (GSH), superoxide dismutase (SOD) and catalase (CAT) levels at *p* < 0.05.

The *Aloe vera* extract (AVE) along with its core ingredients carbohydrate (AVCF) and (PPF) were evaluated by Babu et al. (2021a, b) for managing diabetes in 36 STZ-induced diabetic male wistar rats for 21 days through proteomic analysis approach. AVCF was extracted via hot water extraction leading to ethanol precipitation, while PPF was purified by using 30% trichloroacetic acid precipitation. The study analyzed that effective doses of AVE (300 mg/kg BW), AVCF (54 mg/kg BW) and PPF (0.45 mg/kg BW) attenuated the fasting blood glucose (FBG) by 73.3%, 74.8% and 64.9%, respectively and boosted the insulin levels by 73.8%, 74% and 70.5%, respectively at *p* < 0.0001 (AVE and AVCF) and *p* < 0.001(PPF). Respective doses of AVE, AVCF and PPF restored the cholesterol levels from 44% to 42.6%, 40.4%, and 33.3%, respectively, triglycerides levels from 60.6% to 59.2%, 58.5% and 52.3%, respectively at *p* < 0.001 (AVE and AVCF) and *p* < 0.01 (PPF) and adiponectin levels from 84.3% to 83.6%, 82.8%, and 81%, respectively at *p* < 0.0001 (AVE and AVCF) and *p* < 0.001 (PPF), leading to the improvement in lipid metabolism. AVE and PPF also downregulated the zonulin levels at *p* < 0.0001 but AVCF effect was negligible. Moreover, AVE and AVCF effectively mitigated the hemopexin levels at *p* < 0.001 which led to increase the anti-oxidant enzymes.

Anti-diabetic potential of ethanolic extract of *Aloe vera* was explored by Madhavan et al. (2020) on adipocytes (3T3-L1 cells) using a dosage of 700 µg for 14 days. Ethanolic Soxhlet extraction pursued by rotary evaporation and vacuum dessication was carried to get the extract which was found to be rich in a variety of active constituents, including flavonoids, tannins, and polyphenols, which contributed to stimulate insulin action, strengthen glucose uptake, and decline inflammatory mediators, TNF-*α* and IL-6. Moreover, it also preferably enhanced the adiponectin protein, essential for glucose and fat metabolism, at *p* < 0.05. Additionally, the extract demonstrated high cell viability, non-toxicity, and facilitated cell growth.

Abubakar et al. (2022) reported the protective effects of *Aloe vera* gel (doses of 10% and 20%) against obesity, diabetes, and liver damage in 20 male rats of weight 18–21 gtreated with a high-fat and high-fructose diet (HFFD) for 10 weeks. *Aloe vera* gel was taken in the form of semisolid, directly from rind of plants on daily basis. *Aloe vera* decreased the cholesterol and bad cholesterol (LDL) levels and also facilitated liver health by lowering alanine aminotransferase (ALT) levels and raising albumin levels at *p* < 0.05, but it had an insignificant effect on the good cholesterol (HDL) levels. In addition, it also reduced the oxidative stress in HFFD by boosting the enzymes catalase and SOD levels at *p* < 0.05. Microscopic analysis revealed that a 20% dose of *Aloe vera* significantly sustained the liver composition and structure by inhibiting steatosis (fat deposits). While the 10% dose was more potent in improving the lipid profile and boosting the antioxidant activity.

Afrin et al. (2021) evaluated the hypoglycemic potential of soluble dietary fiber (SDF) isolated from *Aloe vera* and *Abelmoschus esculentus* (Okra) by using 6 STZ-induced (90 mg/kg BW) type 2 diabetes mellitus adult Long Evans rats. The SDF was extracted via the enzymatic hydrolysis method, precipitating in ethanol and then introduced in rats via intestinal perfusion techniques by using a dose of 1.25 g/kg at the rate of 0.5 mL/min for 30 min. After the treatment session, the percentage glucose absorption of *Aloe vera* and okra were 52.98 ± 5.67 (*p* = 0.061) and 57.74 ± 4.81 (*p* = 0.145) against the standard control of 67.74% at *p* < 0.05. *Aloe vera* exhibited a statistically significant reduction in glucose absorption, emphasizing its potential for managing postprandial glycemic control in type 2 DM, while okra was not statistically significant.

Deora et al. (2021) explored the potential of *Aloe vera* gel as an antidiabetic agent via pancreatic cell function and lipid regulation by using 30 STZ-induced diabetic WNIN/GR-OB rats by giving an oral dose of *Aloe vera* gel (300 mg/kg BW) for 28 days. *Aloe vera* gel was isolated by the soxhlet extraction with ethanol and rotary distillation of lyophilized material of *Aloe vera*. *Aloe vera* gel contributed in efficient decline of fasting blood glucose levels (*p* < 0.05) and raised endogenous insulin levels, but not much more significant statistically than standard control. In addition, the gel effectively improved the *β*-cell function (HOMO-*β*) and lowered the insulin resistance (HOMA-IR) at *p* < 0.05. However, after the gel treatment, improvement in *β*-cell integrity and restoration of structure were analyzed via scanning electron microscopy (SEM), emphasizing its protective role. It also decreased the triglycerides, LDL levels, and the TG:HDL ratio at *p* < 0.05 against the standard drug Sitagliptin (*p* < 0.001). Moreover, the gel also acted as a moderate DPP-4 inhibitor as compared to standard sitagliptin, promising *Aloe vera* as a natural remedy for managing diabetes mellitus.

*Aloe vera* and _L_-carnosine were encompassed in a tiny nanophytosome at a formula-based 25:1 ratio by using anti-solvent precipitation techniques, as reported by Darvishi et al. (2021) exhibiting the glycation inhibition activity, revealing a potential solution for microvascular complications like angiogenesis inhibition triggered by a toxic compound, methylglyoxal (MGO) in type 2 diabetes mellitus. This nanophytosome manifested a significant protection of human umbilical vein endothelial cells (HUVECs) subjected to MGO by improving the cell viability by 27 ± 0.5% as compared to other combinations (12–18%) at *p* < 0.0001. It also inhibited the cell death rate in MGO with EC_50_ values of 17.3% (24 h), 15.8% (48 h), and 12.4% (72 h) in a time-dependent manner. At a dose of 500 µg/mL, nanophytosome improved the antioxidant activity and endothelial health by reducing the free radical levels from 83 ± 5 RFU (physical combo) to 63 ± 4 RFU and raising nitric oxide levels from 5.1 ± 0.33 (physical combo) to 26.11 ± 0.19 at *p* < 0.0001. This formulation also exhibited strong pro-angiogenic effects as revealed by improved tube formation (from 2 ± 0.3 to 15 ± 3 tubes), wound healing rate (from 3.07 ± 0.3 mm/h to 4.92 ± 0.3 mm/h), transwell migration (from 394 ± 18 to 586 ± 32), and cell invasion (from 115 ± 5 to 172 ± 9) at *p* < 0.0001. In addition, the HUVECs also exhibited a significant promotion of angiogenic gene expression, including HIF-1*α*, VEGFA, bFGF, KDR, and Ang II, to assure the vascular healing and regeneration.

Desai et al. (2024) studied the antidiabetic effect of combined *Aloe vera* and Glibenclamide (1.63 mg/kg + 0.16 mg/kg) among 30 STZ-induced diabetic male wistar albino rats administered orally for 35 days *Aloe vera* was extracted by the ethanolic aceration followed by rotary vacuum drying techniques. This combination effectively reduced the blood sugar level, body weight, and HbA1c from 317.0 ± 9.88 mg/dL to 129.5 ± 7.21 mg/dL, 242.2 ± 4.32 g to 208.6 ± 5.33 g, and 5.3 ± 0.35% to 4.9 ± 0.41%, respectively, at *p *< 0.05. It also declined the total cholesterol from 187.5 ± 3.55 mg/dL to 155.2 ± 3.13 mg/dL, LDL levels from 100.4 ± 2.89 mg/dL to 69.1 ± 2.82 mg/dL and raised the HDL levels from 40.03 ± 3.41 mg/dL to 53.67 ± 2.43 mg/dL, leading to regulate lipid metabolism. It also reduced the oxidative stress by enhancing the concentration of GSH from 394.8 ± 32.98 mol/g to 851.66 ± 33.47 mol/g and lowering the MDA levels from 904.85 ± 42.32 mol/g to 601.72 ± 43.63 mol/g at *p* < 0.05.

#### Clinical studies related to metabolic regulation

Prakash et al. (2023) studied the anti-diabetic properties of processed *Aloe vera* juice (highly refined product) on 60 Indian diabetic patients of type 2 diabetes mellitus along with demographic factors of various stages administered orally with a dose of 20 ml for 30 days. Out of 4 groups, the blood sugar level of two groups was regulated by anti-diabetic medication (standard drugs), and the other two groups with *Aloe vera* juice. Results showed that sugar level was preferably decreased from 177.43 ± 17.64 mg/dL to 128.76 ± 27.50 mg/dL at t-value 13.15 in *Aloe vera* juice-treated patients. In comparison to control group, insignificant decrease in blood sugar level was observed, as it was regulated from 170.96 ± 18.34 mg/dL to 172.26 ± 17.64 mg/dL at t-value of 1.22.

The study was performed by Budiastutik and Ningsih (2023) to interpret the impact of *Aloe vera* juice on reducing fasting glycemia among 12 pre-diabetic patients. Thermal-supported gel extraction, organoleptic improvement by flavors and blending processes was used to get effective *Aloe vera* juice. The consumption of 175 mL of *Aloe vera* juice for 15 days led to significant fall in fasting blood glucose from 107.4 mg/dL to 92.1 mg/dL at *p* < 0.001.

Fandizal et al. (2021) evaluated the efficiency of *Aloe vera* and cinnamon drink in lowering blood glucose in 60 type-2 diabetes mellitus patients via non-equivalent quasi-experimental manner. Cinnamon extracted by thermal techniques and direct addition of isolated *Aloe vera* gel in cinnamon was done to make drink. *Aloe vera* (0.8 g/kg BB) and cinnamon (10 g/100 cc) water solution effectively declined the blood glucose levels by 153 mg/dl and 107 mg/dl, respectively at *p* < 0.05 after 24 h.

Younis et al. (2022) studied the comparative effect of glycemic control of dried *Aloe vera* powder (10, 15 and 20 mg) extracted by aqueous extraction, in type-1 and type-2 DM among 15 patients (7 with type-1 DM and 8 with type-2 DM) for 28 days. In type-1 diabetic patients, *Aloe vera* had weak influence, with blood glucose levels exhibiting minimal change from 261 ± 20.41 mg/dL to 243 ± 7.67 mg/dL (10 mg dose), 191 ± 8.01 mg/dL to 144 ± 13.27 mg/dL (15 mg dose) and 204 ± 8.82 mg/dL to 137 ± 6.82 mg/dL (20 mg dose) at *p* ≤ 0.05. While in type-2 diabetic patients, it significantly suppressed the blood glucose levels from 194 ± 7.52 mg/dL to 114 ± 6.96 mg/dL (potent dose of 10 mg), 188 ± 5.95 mg/dL to 135 ± 5.25 mg/dL (15 mg dose), and 179 ± 4.76 mg/dL to 161 ± 3.55 mg/dL (20 mg dose) at *p* ≤ 0.05. Results exposed that *Aloe vera* was more potent against type-2 DM than type-1 DM patients. The study also emphasized the curative effect of the *Aloe vera* powder, containing 0.21 mg (QE)/g flavonoids and 2.4 mg (QE)/g phenolic compounds which are prominent for their potent antioxidant and anti-inflammatory activity. *Aloe vera* could be used as a supplement but unable to mitigate the lipid oxidative degradation completely.

Aisya et al. evaluated the anti-diabetic efficacy of *Aloe vera*-derived drink in 38 participants (of age 35–36 years) given an oral dose of 165 g/day for 4 weeks. *Aloe vera* leaf latex was extracted from leaf, cut into 1 cm^3^ cube, mixed with citrate and then washed and stored after 10 min. The *Aloe vera* treated groups had a statistically effective reduction of glycated albumin from 19.3 ± 1.73% to 14.9 ± 2.22% at *p* < 0.001 and HOMA-IR from 3.6 ± 2.04 to 1.9 ± 1.13 at *p* = 0.001 against standard control. *Aloe vera* gel was efficacious and non-toxic, but the limited sample size and time duration limited its broad generalizability.

**Fig. 8:**
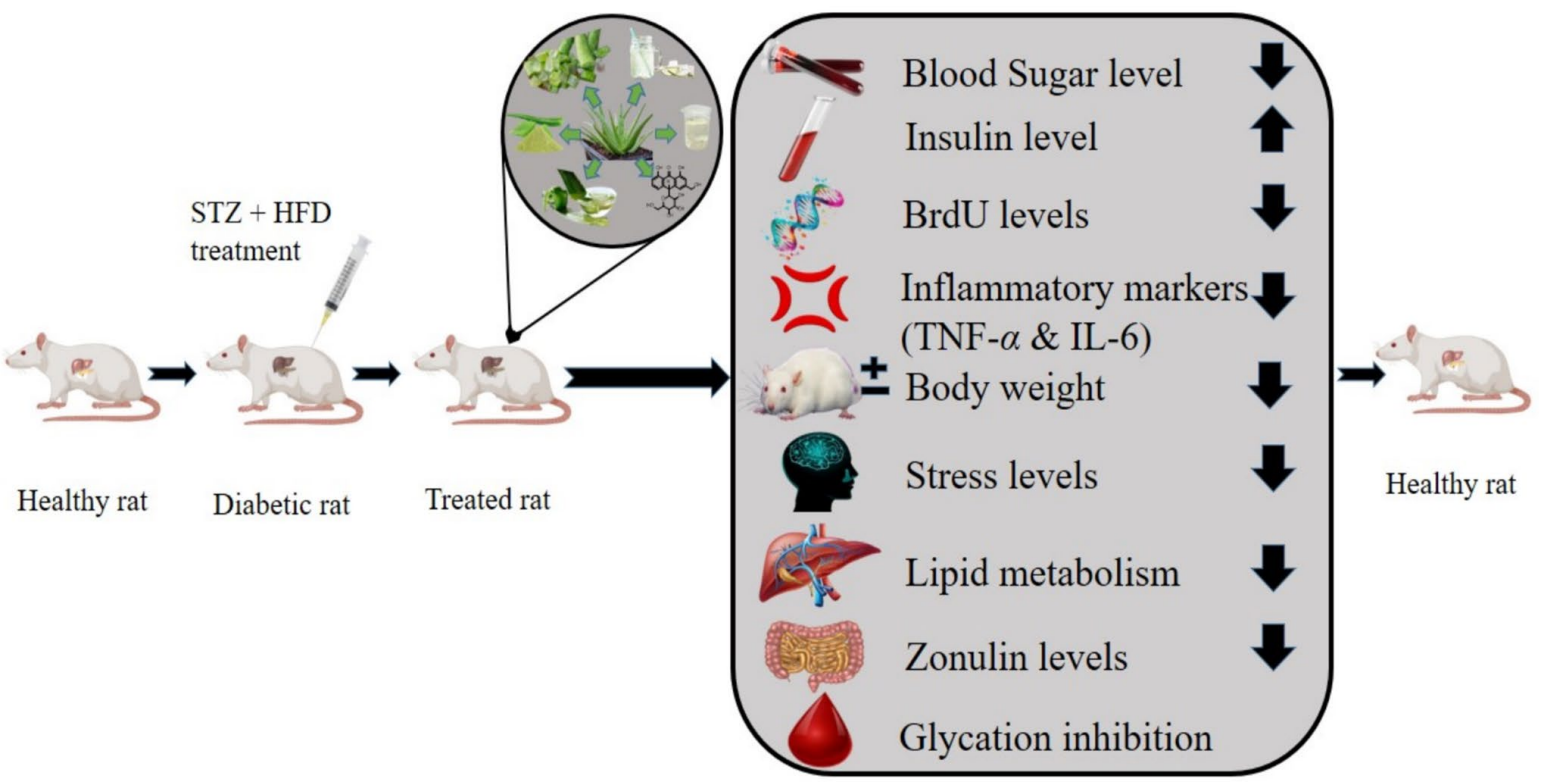
Metabolic regulation of biophysiological parameters through *in-vivo* study

*Aloe vera* with its extracts and derived active components had been proved to be an effective potential against diabetes mellitus. *Aloe vera’s* extracts and its ingredients were given to diabetic rats to observe the changes via metabolic parameters like blood glucose and insulin levels along with BrdU levels, inflammatory markers, body weight, stress markers, lipid profile levels and zonulin levels promised *Aloe vera* as not only antidiabetic remedy but also anti-inflammatory, antioxidant and lipid homeostasis activities that is beneficial for diabetic health (Fig. 8). A summarized Table 2 provides variation of results by using different formulation like gel, leaf extract, powder or drink with various dosages and time period, offering consistency of *Aloe vera* in diabetes management.

**Table 2:** Summary of metabolic regulations by *Aloe vera*’s different formulation like gel, leaf extract, powder or drink with various dosages and time period in diabetes management

Formulation type	Sample size	Dosages & frequency	Duration	Key findings	References
*Aloe vera* gel	30 STZ-induced diabetic female Albino rats	Orally, 300 mg/kg	21 days	Blood glucose level: 250 mg/dL to 113 mg/dL Cholesterol level: 97 mg/dL to 71 mg/dL Triglycerides level: 122 mg/dL to 102 mg/dL	Hasan and Abdullah (2022)
	24 alloxan monohydrate male Albino rats	Orally, 1000 mg/kg	21 days	Glucose level: 254.64 ± 1.50 mg/dL to 93.80 ± 0.60 mg/dL Live body weight: 118.88 ± 0.60 g to 161.10 ± 0.74 g	Fazlani et al. (2020)
	20 male rats (weight 18–21 g)	Doses of 10% and 20%	10 weeks	Decreased Cholesterol, LDL levels, oxidative stress and increase albumin level at *p* < 0.05 Inhibit steatosis (potent 20%) Improve lipid profile (potent 10%)	Abubakar et al. (2022)
	30 STZ-induced diabetic WNIN/GR-OB rats	Orally, 300 mg/kg	28 days	Effective lowering of fasting blood glucose, HOMA-IR, triglycerides, LDL levels, TG:HDL ratio at *p* < 0.05 Increase the endogenous insulin levels, HOMO-*β*, *β*-cell integrity at *p* < 0.05	Deora et al. (2021)
*Aloe vera* juice	30 STZ-induced female white *Rattus norvegicus* rats	Orally, 1.8 ml/200 g	12 days	Blood glucose level: 498.60 ± 89.77 mg/dl to 77.4 ± 8.89 mg/dl Live body weight: 150.30 ± 10.41 g to 167.4 ± 13.37 g	Ramadhini and Ritonga (2022)
*Aloe vera* dried powder	25 STZ-induced diabetic male sprauge-dawley rats	1.5 g, 3.0 g, 4.5 g	24 days	Lowered the thyroid hormones: Triiodothyronine at *p* = 0.12, thyroxine at *p* > 0.05, and cortisol level at *p* = 0.00	Fatima et al. (2024)
	40 STZ-induced T2DM sprauge dawley rats	Orally, 300 mg/kg	60 days	Fasting blood sugar: 498.40 mg/dl to 89.30 mg/dl HbA1c: 11.84% to 4.02% at *p* < 0.001	Javaid and Waheed (2020a)
	40 STZ-induced T2DM sprauge dawley rats	Orally, 300 mg/kg	60 days	Triglycerides level: 221.00 mg/dl to 112.50 mg/dl	Javaid and Waheed (2020b)
	HUVECs cells	500 µg/mL	72 h	Improve cell viability: 27 ± 0.5% at *p* < 0.0001 Cell death rate: EC_50_ = 17.3%(24 h), 15.8%(48 h), and 12.4%(72 h) Free radical levels: 83 ± 5 RFU to 63 ± 4 RFU Nitric oxide levels: 5.1 ± 0.33 to 26.11 ± 0.19 Tube formation: 2 ± 0.3 to 15 ± 3 tubes Wound healing rate: 3.07 ± 0.3 mm/h to 4.92 ± 0.3 mm/h Transwell migration: 394 ± 18 to 586 ± 32 Cell invasion: 115 ± 5 to 172 ± 9 at *p* < 0.001	Darvishi et al. (2021)
*Aloe vera* extract + ingredients	26 STZ-induced albino rats (weigh 200–250 g)	30 mg/kg	30 days	Lowered the glucose level, MDA, GSH, SOD, CAT levels and elevated the insulin levels at *p* < 0.05	Al-Sowayan and AL-Sallali (2023)
	36 STZ-induced diabetic male wistar rats	AVE (300 mg/kg)	21 days	Reduction of FBG:73.3%; Boosted insulin level:73.8% Cholesterol level: 44% to 42.6% Triglyceride level:60.6% to 59.2% Adiponectin level:84.3% to 83.6%	Babu et al. (2021a, b)
		AVCF(54 mg/kg)		Reduction of FBG: 74.8%; Boosted insulin level:74% Cholesterol level:44% to 40.4%% Triglyceride level:60.6% to 58.5% Adiponectin level:84.3% to 82.8%	
		PPF(0.45 mg/kg)		Reduction of FBG:64.9%; Boosted insulin level:70.5% Cholesterol level:44% to 33.3% Triglyceride level:60.6% to 52.3% Adiponectin level:84.3% to 81%	
	3T3-L1 cells	700 µg	14 days	Decreased the inflammatory markers: TNF-* α* and IL-6; increased the adiponectin protein at *p* < 0.05	Madhavan et al. (2020)
	30 STZ-induced diabetic male wistar albino rats	Orally, 1.63 mg/kg (*Aloe vera*) + 0.16 mg/kg (Glibenclamide)	35 days	Blood sugar level: 317.0 ± 9.88 mg/dL to 129.5 ± 7.21 mg/dL Body weight: 242.2 ± 4.32 g to 208.6 ± 5.33 g HbA1c: 5.3 ± 0.35% to 4.9 ± 0.41% Total cholesterol: 187.5 ± 3.55 mg/dL to 155.2 ± 3.13 mg/dL LDL levels: 100.4 ± 2.89 mg/dL to 69.1 ± 2.82 mg/dL HDL levels: 40.03 ± 3.41 mg/dL to 53.67 ± 2.43 mg/dL Reduced Oxidative stress: GSH: 394.8 ± 32.98 mol/g to 851.66 ± 33.47 mol/g MDA: 904.85 ± 42.32 mol/g to 601.72 ± 43.63 mol/g at *p* < 0.05	Desai et al. (2024)
*Aloe vera*-derived soluble dietary fibre	6 STZ-induced T2DM adults long evans rats	1.25 g/kg at the rate of 0.5 mL/min	30 min	Glucose absorption: 52.98 ± 5.67 at *p* = 0.061	Afrin et al. (2021)

The enzymatic inhibition effect of *Aloe vera* and its constituents on metabolic parameters like blood glucose, lipid profile, and oxidative stress contributed to the protective actions towards diabetic complications. By regulating metabolic parameters, *Aloe vera* attenuated the emerging pathologies often associated with hyperglycemia, like obesity and renal, neural, and sexual issues. Slow healing of diabetic wounds is a chronic and common complication. Regulation of metabolic parameters ensured the multidimensional efficacy of *Aloe vera* toward prolonged hyperglycemia, which demonstrated its comprehensive antidiabetic potential.

### Role of Aloe vera in diabetic complications

#### Anti-obesity potential

The anti-obesity efficacy of *Aloe vera* gel extract (AVGE) containing *Aloe* sterols was evaluated by Tada et al. (2020) by assessing its impact on brown adipose tissue (BAT) activation. *Aloe vera* gel was extracted by cold ethanol maceration, thermal drying and rotary supported evaporation. The experimental strategy was implemented using 24 male high-fat-diet-treated mice, administered *Aloe vera* gel extract orally at a dose of 0.2 mg/g BW per day for 10 weeks. AVGE-treated rats had significantly reduced the body weight from 41.0 ± 0.5 g to 38.6 ± 0.9 g at *p* < 0.05, regardless of their calorie intake. Fat storage was also declined by decreasing the visceral fat (vWAT) from 1,195.5 ± 93.3 mg to 939.3 ± 97.2 mg, inguinal fat (iWAT) from 2,369.6 ± 126.1 mg to 2,196.3 ± 124.8 mg and liver weight from 1,675.9 ± 126.1 mg to 1,434.7 ± 107.9 mg. At the cellular level, AVGE activated the BAT by enhancing expression of the key thermogenic genes, including Ucp1, Adrb3, and Cidea, at *p* < 0.05. In vitro studies, AVGE significantly exhibited the strong elevation of Fibroblast Growth Factor 21 hormone (FGF21) levels in comparison with bezafibrate, but in vivo, FGF21 expression was not statistically significant.

Javaid and Waheed (2024) demonstrated the *Aloe vera*’s anti-obesity potential contrasted with sitagliptin among 40 STZ-induced type-2 DM male albino rats by giving an oral dose of *Aloe vera* leaf extract (300 mg/kg) for 60 days. *Aloe vera* extract was extracted physically from leafs without any solvent and dried in sunlight. *Aloe vera* leaf extract was significantly effective in obesity mitigation by lowering body mass from 272.00 g to 249.90 g against standard control at *p* < 0.001.

Thilavech et al. (2024) assessed the postprandial metabolic responses of *Aloe vera* juice extracted by centrifugation-based mechanical extraction of Aloe gel and acemannan among 16 obese men given four meals as high-fat (HF) meal, HF with *Aloe vera* juice (AVJ), HF + AV with 0.5 g acemannan, and HF + AV with 1 g acemannan for 4 weeks. The serum triglyceride level was decreased progressively from 109.5 ± 14.8 mg/dL (HF) to 101.7 ± 8.9 mg/dL (HF + AVJ), 99.3 ± 10.1 mg/dL (HF + AV with 0.5 g acemannan), and 94.3 ± 9.2 mg/dL (more potent meal HF + AV with 1 g acemannan) at *p* < 0.05. The serum free fatty acid also declined from 0.76 ± 0.07 mmol/L (HF) to 0.74 ± 0.04 mmol/L (HF + AVJ), 0.61 ± 0.06 mmol/L (more potent meal HF + AV with 0.5 g Ace), and 0.71 ± 0.05 mmol/L (HF + AV with 1 g Ace) at *p* < 0.05. The consumption of *Aloe vera* juice and acemannan (both doses of 0.5 and 1 g) significantly reduced the plasma glucose and inflammatory mediator TNF-*α* at *p* < 0.05.

Yunusoglu et al. (2022) evaluated *Aloe vera* as a natural remedy with potential anti-obesity properties by applying different doses of *Aloe vera* extracts isolated by the heat-based ethanolic extraction with rotary evaporation methods ranging from 10–100 µg/mL on 3T3-L1 cells, focusing on inhibiting the adipogenesis mechanism. Findings revealed that lower dosages of *Aloe vera* (10–50 µg/mL) showed no cytotoxicity on cells, having an IC_10_ value of 43.83 µg/mL. The potent dosages (30–50 µg/mL) down regulated the cytotoxicity by *p* < 0.05, identified via lactate dehydrogenase (LDH) release assay. *Aloe vera* extracts exhibited a reduction in intracellular triglyceride levels by treating with 40 and 50 µg/mL doses, and glycerol-3-phosphate dehydrogenase (GPDH) enzymes with 30–50 µg/mL doses in a dose-dependent manner at *p* < 0.05. In addition, gene expression levels involving fat metabolism were decreased in *Aloe vera* treated cells like PPAR*γ*, SREBP-1c, FAS and aP2 levels with 40–50 µg/mL doses, C/EBP*α*, HSL and ATGL levels with 30–50 µg/mL and ACC levels with 20–50 µg/mL respectively, leading to smaller size of cell at *p* < 0.05. However, it showed a mild toxicity at higher concentration (more than 60 µg/ml).

#### Nephroprotective potential

**Fig. 9:**
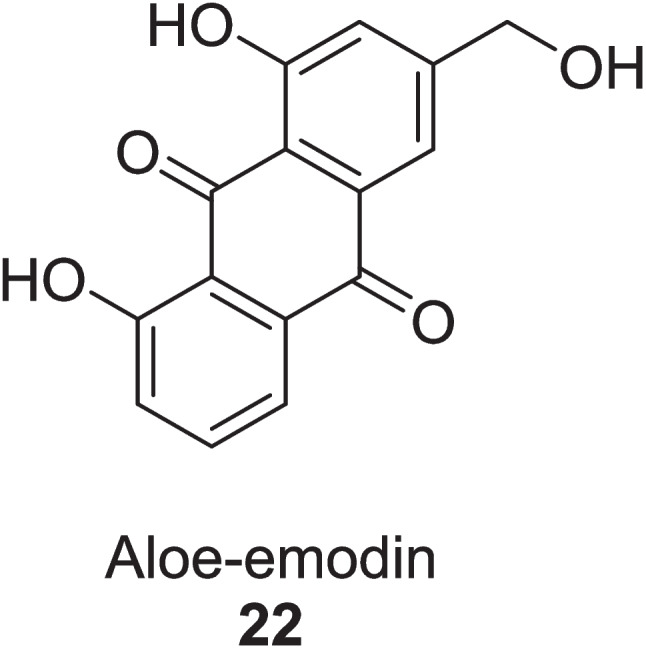
IRF4 Inhibitor extracted from *Aloe vera*

Lu and Li (2022) reported the remedial competency of *Aloe vera*’s constituent compound **22** in diabetic nephropathy by inhibition of interferon regulatory factor 4 (IRF4) having IC_50_ value of 8.038 µM in human podocytes cells. It also inhibited the IRF4 to lower the inflammatory mediators like IL-13 and IL-7, collagen I levels (causing fibrosis and scarring) and deactivation of Notch1 and p-AKT pathways (Fig. 9). In vivo studies, a dosage of 20 mg/kg per day was administered orally to 18 STZ-induced diabetic Sprauge-Dawley rats for 10 weeks reflected improvement in renal filtration by reducing inflammation, urinary albumin, urinary creatinine, urinary albumin to creatinine ratio and blood urea nitrogen at *p* < 0.01.

Santos et al. (2021) reported the impact of *Aloe vera* butanolic extract (AVB) to fortify against diabetic nephropathy via a proteomic approach in 27 STZ-induced (55 mg/kg BW) T1DM male wistar rats by giving a dose of 50 mg/kg per day orally for 20 days. The AVB was extracted by partition solvent extraction, suppressed glycemia within 6 h at *p *< 0.05. It also improved the mitochondrial efficiency, vascular integrity, and glucose metabolism by regulating the 9 key proteins i.e., chaperonin 10, cytochrome P450 2C23, aldolase A, transaldolase, cAMP-dependent protein kinase catalytic subunit beta, arginase 2, aldolase A, and D-lactate dehydrogenase, contributing protection against kidney damage.

Şeker et al. (2023) evaluated the renal-protective potential of *Aloe vera *via oxidative stress and apoptotic activity among 21 female STZ-induced diabetic rats, given daily dose of 300 mg/kg for 14 days. *Aloe vera* was extracted via ethanolic extraction. *Aloe vera* extract regulated the oxidative stress by lowering the malondialdehyde from 21.39 ± 8.37 nmol/gm to 12.29 ± 3.74 nmol/gm and reactive oxygen species from 60.67 ± 10.96 rlu/mg to 54.53 ± 8.12 rlu/mg at *p* < 0.05. Apoptotic activity was significantly reduced by reducing the bax and caspase proteins from 30.50 ± 5.13% to 27.57 ± 3.37% and 32.91 ± 8.79% to 28.24 ± 3.93%, respectively at *p* < 0.05. Moreover, it also alleviated the apoptotic index from 10.79 ± 3.71% to 6.40 ± 2.27%, analyzed via TUNEL staining at *p* < 0.05. Moreover, aquaporin 3 (AQP3) protein in the cortex was restored from 49.02 ± 12.34% to 57.64 ± 9.22% at *p* < 0.05, while the medulla AQP3 had an insignificant effect.

The nephro and neuroprotective potential of *Aloe vera* was studied by Dibal et al. (2024) in 30 ethanol-induced male rats, administered the methanol extract (ME) (dosages of 50 and 100 mg/kg) and aqueous extract (AE) (25 and 50 mg/kg) of *Aloe vera* for 18 days. Extraction of *Aloe vera* was done by solvent extraction using methanol in ratio 1:1. *Aloe vera* showed significant improvement in renal function by lowering the creatinine levels from 0.41 mg/dL to 0.40 mg/dL (100 mg/kg ME) and 0.36 mg/dL (50 mg/kg AE), and urea levels to 33.52 mg/dL (100 mg/kg ME) to 25.11 mg/dL (50 mg/kg AE), respectively, at *p* < 0.05. It also alleviated the malondialdehyde (MDA) levels from 9.54 mg/dL to 6.70 mg/dL (100 mg/kg ME) and 7.05 mg/dL (50 mg/kg AE), respectively, and enhanced the catalase activity from 7.98 mg/dL to 10.43 mg/dL (100 mg/kg ME) and 10.98 mg/dL (50 mg/kg AE) and glutathione activity from 5.62 to 7.98 (50 mg/kg ME) and 7.91 mg/dL (50 mg/kg AE), respectively, at *p* < 0.05, leading to the promotion of brain health. The microscopic analysis revealed that *Aloe vera* extracts facilitated the reduction of neuron degeneration in both cerebellum and cerebrum.

#### Neuroprotective potential

Malayeri et al. (2021) investigated the protective effect of *Aloe vera* combined with aerobic exercise against type 2 diabetic nerve complications among 32 female diabetic patients of age 40–55 years by giving *Aloe vera* (400 mg/kg) orally for 8 weeks. *Aloe vera* significantly decreased the blood sugar from 10.94 ± 0.929 mmol/L to 8.48 ± 0.749 mmol/L, insulin levels from 7.46 ± 5.65 mU/mL to 5.77 ± 0.400 mU/mL, insulin resistance from 3.64 ± 0.587 to 2.18 ± 0.340, body weight from 29.17 ± 1.91 kg/m^2^ to 26.26 ± 2.38 kg/m^2^ and body fat percentage from 33.88 ± 1.65% to 30.27 ± 1.66% at *p* ≤ 0.5. In addition, it also significantly enhanced the brain-derived neurotrophic factor (BDNF) from 160.5 ± 2.81 ng/L to 215.9 ± 9.39 ng/L, insulin sensitivity from 0.525 ± 0.019 to 0.592 ± 0.024 and aerobic capacity 26.13 ± 1.14 mL/kg/min to 28.27 ± 2.01 mL/kg/min at *p* ≤ 0.5.

Mahabady et al. (2021) showed the neuroprotective effect of *Aloe vera* gel by evaluating the nerve growth factor (NGF) and neurotrophin receptor signaling pathways among 25 STZ-induced male Wistar rats. *Aloe vera* gel was purchased by commercial source Barij Essence Pharmaceutical Company, Iran. Over the period of 8 weeks, these rats were given *Aloe vera* gel of 400 mg/kg/day orally. *Aloe vera* gel significantly elevated the body weight from 205.7 ± 4.2 mg/dl to 248.3 ± 8.6 mg/dl and alleviated the blood glucose level from 536.7 ± 26.6 mg/dL to 255.4 ± 32.5 mg/dL at *p* < 0.05. In the context of neuropathy, NGF in *Aloe vera*-treated groups was lowered from 8.48 ± 0.40 to 4.34 ± 0.47 against the reference control. However, the tropomyosin receptor kinase A (TrkA) were increased and p75 receptors were decreased significantly against the standard control at *p* < 0.05.

Omer et al. (2023) provided the full depth detail of the neuroprotective action of *Aloe vera* ingredient, barbaloin administered orally (doses of 25 and 50 mg/kg) in 30 STZ-induced male diabetic rats for 30 days. The barbaloin effectively reduced the blood sugar level from 106.42 ± 3.00 mg/dL to 270.7 ± 6.58 mg/dL (25 mg/kg) and 97.25 ± 3.57 to 217.6 ± 2.51 mg/dL (50 mg/kg) compared to standard which is 295.6 ± 6.78, and maintained the body weight from 219.21 ± 3.24 g to 215.8 ± 5.58 g (25 mg/kg) and 219.15 ± 3.36 g to 219.2 ± 5.62 g (50 mg/kg) at *p* < 0.0001. The higher potent dose significantly declined the oxidative stress by lowering the malondialdehyde (MDA) from 7.5 nmol/mg to 5.0 nmol/mg (25 mg/kg) and 3.5 nmol/mg (50 mg/kg) and increasing SOD, CAT, and GSH levels from 3.0 U/mg to 4.5 U/mg (25 mg/kg) and 5.5 U/mg (50 mg/kg) for all parameters at *p* < 0.0001. The cholinergic dysfunction markers were regulated by elevating the choline acetyltransferase (ChAT) enzymes from 100 U/g to 125 U/g (lower dose) and 175 U/g (higher dose) and alleviating the acetylcholinesterase (AChE) enzymes from 7.5 µmolAcSCh/min/mg to 6.0 µmolAcSCh/min/mg and 5.0 µmolAcSCh/min/mg at *p* < 0.0001. Moreover, it also enhanced the learning and memory ability of rats, analyzed by the Y-maze and Morris water maze tests. The higher potent dose also exhibited the significant reduction of the neuroinflammatory mediators like NF-*κ*B from 35 ng/mg to 15 ng/mg, IL-1*β* from 60 pg/kg to 35 pg/kg, IL-6 from 80 pg/mg to 40 pg/mg, and TNF-*α* from 150 pg/mL to 75 pg/mL, contributing a strong anti-inflammatory effect.

#### Sexual health

Ghaffari et al. (2023) reported the beneficial impact of *Aloe vera* extracted by hydroalcohlic maceration method, in shielding against the diabetic sexual complications among 12 STZ-induced male diabetic wistar rats that were administered *Aloe vera* gel (380 mg/kg) for 30 days. *Aloe vera* gel significantly alleviated the blood glucose sugar from 528.8 ± 27.41 mg/dL to 214.2 ± 14.37 mg/dL and elevated the body weight from 195.6 ± 5.98 g to 217.8 ± 17.06 g, respectively at *p* < 0.05. Testosterone levels were upregulated significantly from 2.32 ± 0.44 ng/dL to 3.19 ± 0.63 ng/dL. Sperm morphology revealed that *Aloe vera* gel enhanced the counts of sperm (138.2 ± 5.21 n/mm^2^ to 169 ± 6.1 n/mm^2^), Sertoli cells (1.2 ± 0.2 n/mm^2^ to 1.6 ± 0.2 n/mm^2^), spermatogonia cells (78.4 ± 4.2 n/mm^2^ to 84.8 ± 3.51 n/mm^2^), Leydig cells (7.2 ± 0.58 n/mm^2^ to 7.8 ± 0.37 n/mm^2^), and highly motile sperm (3.6 ± 1.12% to 7.4 ± 2.51%) at *p* < 0.05. The diameter of the seminiferous tubule and germinal epithelium layer were significantly ameliorated from 229.45 ± 6.22 µm to 255.06 ± 6.22 µm and 16 ± 1.26 µm to 19 ± 1.26 µm, respectively at *p* < 0.05.

Dibal et al. (2023) reported the protective effect of *Aloe vera* among 20 BALB/c rats fed with a rich-fat and high-fructose diet (FRHFD) by giving the doses of 10 g and 20 g of *Aloe vera* gel taken freshly from the *Aloe vera* in addition to FRHFD for 10 weeks. Blood glucose level was statistically lowered by both doses (10 g and 20 g) against the FRHFD-treated groups. Microscopic findings revealed that *Aloe vera* gel (more potent at lower doses) shielded the pancreas by retaining acini, islet cells and the spleen by reviving lymphoid cells. In addition, it also improved the testicular health by facilitating sugar metabolism, Leydig cells, and spermatogonia cells at *p* < 0.05.

**Fig. 10:**
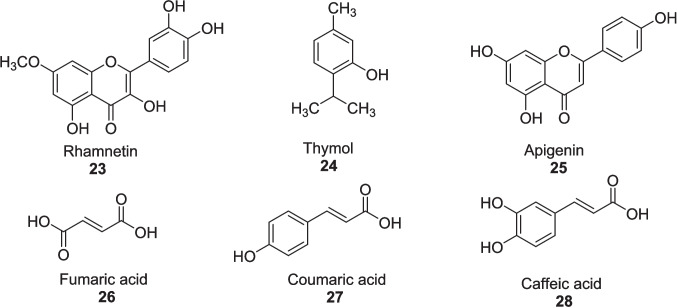
Bioactive compounds from *Aloe vera* hydro-ethanol extract

**Fig. 11:**
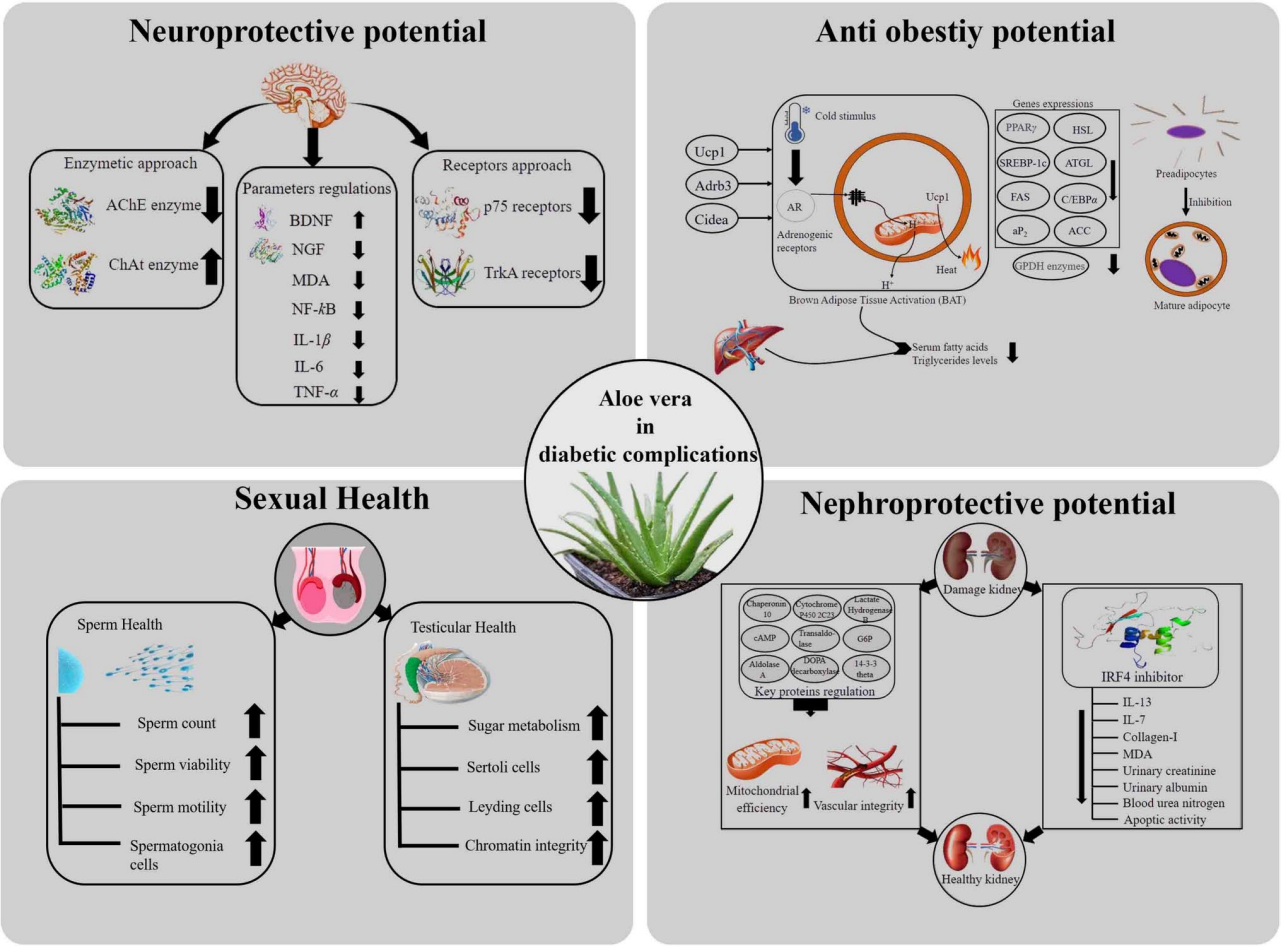
Mechanistic representations of role of *Aloe vera* in treating diabetic complications

Pal et al. (2024) evaluated the efficacy of *Aloe vera* hydro-ethanol extract (AVHE) to protect from sperm damage via an oxidative stress approach in 32 male diabetic humans and wistar rats after giving the different doses of AVHE (1 mg/mL, 2 mg/mL and 4 mg/mL) for 28 days. Gel from leaves of *Aloe vera* was processed with hydro-ethanol (ratio of 1:2) for 48 h to get AVHE. *In vitro* studies, AVHE treatment of 1 mg/mL, 2 mg/mL, and 4 mg/mL significantly improved the sperm motility from 33.03 ± 0.84% to 50.34 ± 0.64%, 50.87 ± 0.43%, and 51.33 ± 0.75% in humans, respectively, and from 24.57 ± 0.42% to 40.72 ± 0.45%, 41.23 ± 0.57%, and 41.40 ± 0.70% in rats, respectively. Respective doses enhanced sperm viability by 50.34 ± 0.64%, 50.87 ± 0.87%, and 51.33 ± 0.75% in humans and 40.72 ± 0.45%, 41.23 ± 0.57%, and 41.40 ± 0.70% in rats, which was dropped from 85.91 ± 1.91% to 33.78 ± 1.33% (humans) and 87.28 ± 0.62% to 28.75 ± 0.50% (rats). Deoxyribonucleic acid (DNA) fragmentation was reduced by elevating the chromatin integrity to 62.95 ± 0.31%, 62.25 ± 0.23%, and 63.43 ± 0.23% in humans and 61.42 ± 0.41%, 61.89 ± 0.89%, and 62.23 ± 0.59% in rats. Plasma membrane integrity was stimulated by 36.36 ± 0.95%, 36.13 ± 0.46%, and 37.60 ± 0.62% in humans and 26.48 ± 0.35%, 27.61 ± 0.45%, and 28.620 ± 41% in rats. Sperm apoptosis was notably reduced to 20.25%, 10.72%, and 9.34% in humans and 16.02%, 14.37%, and 7.80% in rats as analyzed via the terminal deoxynucleotidyl transferase dUTP nick end labeling (TUNEL) test. AVHE exhibited the inhibition of DPPH with an IC_50_ value of 34.21 ppm and raised the activity of SOD by 23.59%, 23.79%, and 23.92% (humans) and 36.12%, 39.16%, and 39.54% (rats) and catalase enzyme by 42.87%, 43.48%, and 45% (humans) and 7.06%, 9.44%, and 8.83% (rats), while TBARS levels were decreased by 4.44%, 6.26%, and 6.57% (humans) and by 7.06%, 9.44%, and 8.83% (rats). Phyto-compounds **23, 24, 25, 26, 27** and **28** found in AVHE were identified via LC–MS test (Fig. 10). As a whole, *Aloe vera* also found beneficial in diabetic complications (Fig. 11).

As a whole, *Aloe vera* is found beneficial in diabetic complications including anti-obesity by lowering the live body weight and fat storage in cells, nephroprotective by IRF4 inhibition, kidney-related parameters like inflammatory mediators, creatinine and urea levels, neuroprotective via enzymatic and recpetors approach along with regulation of BDNF, NGF, MDA and inflammatory markers, and also sexual health by improving the sperm health (motility, counts, viability) and testicular health (Fig. 11).

#### Wound healing potential

Pichaivel et al. (2021) reported the therapeutic influence of *Aloe vera* in wound healing among 18 STZ-induced diabetic wistar rats via tissue regeneration and biochemical pathways. These rats were given 50 mg/kg/day of *Aloe vera* gel extracted by the aqueous extraction followed by thermal drying, orally for 10 days. *Aloe vera* significantly enhanced tissue formation by boosting the dry granulation tissue weight from 22.5 ± 4.50 mg to 36.5 ± 4.60 mg, wet granulation weight from 169.5 ± 10.32 mg to 270.5 ± 12.09 mg, tissue tensile strength from 176.36 ± 1.10 g to 317.43 ± 15.07 g, hydroxypyroline tissue from 12.18 ± 3.20 mg/g to 14.74 ± 4.62 mg/g, and lysyl oxidase enzyme from 1129 ± 46 SFU to 1910 ± 61 SFU at *p* < 0.01. In the context of biochemical pathways, it enhanced the tissue proteins from 25.5 ± 2.60 mg/g to 41.18 ± 4.10 mg/g and glycosaminoglycan synthesis by elevating hexuronic acid from 9.2 ± 1.12 mg/g to 15.21 ± 3.19 mg/g and hexosamine 7.4 ± 1.40 mg/g to 11.39 ± 2.47 mg/g, demonstrating its potential in wound healing.

A biocellulose (BC) sheet incorporated with *Aloe vera* gel (BC/AV) via passive soaking and thermal drying, constituting 12.32 ± 3.4% protein and having a molecular weight of 20 kDa, was formulated by Yosboonruang et al. (2023) to enhance the healing activity of diabetic wounds in 15 male sprauge-dawley rats. The BC/AV sheet exhibited water absorption of 74% and anti-inflammatory activity by lowering TNF-*α* levels effectively proportional to doses. The therapeutic potential of the BC/AV sheet was evaluated via protein diffusion of 86.765 ± 10.85% (2 h) and 97.23% (4 h). In vivo study, the diabetic wound was healed in 14 days using the BC/AV sheet, much faster than negative group.

**Fig. 12:**
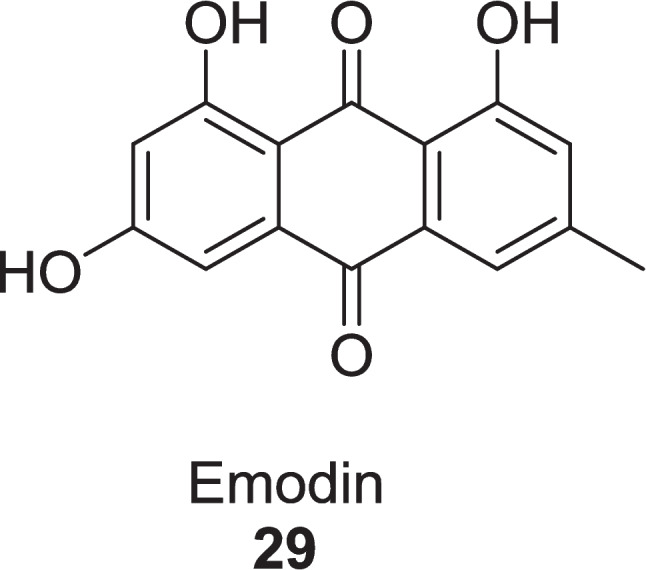
TGF-*β*1 inhibitors from *Aloe vera*

Roney et al. (2024a) evaluated the major phytoconstituents of *Aloe vera* as an alternative approach to diabetic wound healing via the TGF-*β*1 inhibition mechanism through in silico study. The binding energies of active compounds, Aloe-emodin **22**, Aloin **13**, Aloesin **21**, and Emodin **29** were −9.6 kcal/mol, −9.1 kcal/mol, −8.7 kcal/mol, and −9.6 kcal/mol, respectively, identified via *in silico* analyses (Fig. 12). While compound **22** was more potent TGF-*β*1 inhibitor, having high binding affinity (−31.03 ± 3.57 kcal/mol), high intestinal absorption (74.17%), high probability of activity (pa > 0.7), and low blood brain barrier permeability of −0.72. Compound **22** followed Lipinski’s rule of five and had an LD_50_ value of 5000 mg/kg, promising to be a good and safe drug. Molecular dynamic simulation of the TGF-*β*1 complex at 200 ns revealed its stability and flexibility with root mean square deviation (1.69 ± 0.27 Å), root mean square fluctuation (14.97 ± 5.02 Å), radius of gyration (19.74 ± 0.07 Å), and solvent accessible surface area (13,980.90 ± 323.52 Å^2^) identified via Amber18 software.

**Fig. 13:**
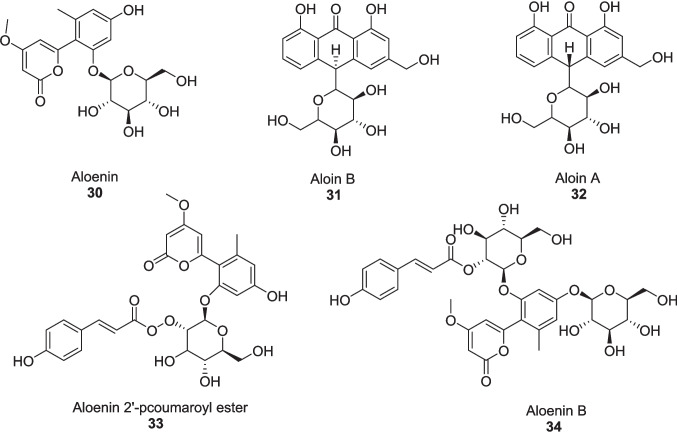
GSK3-*β* Inhibitors from *Aloe vera*

Roney et al. (2024b) extracted and evaluated the six active compounds from *Aloe vera* leaves as a glycogen synthase kinase-3*β* (GSK3-*β*) inhibitor to treat wound healing in diabetic conditions, using in silico techniques. The binding energies of compounds **30**, **22**, **31**, **32, 33**, and **34** were −7.6 kcal/mol, −7.8 kcal/mol, −8.1 kcal/mol, −8.6 kcal/mol, −9.5 kcal/mol, and −8.4 kcal/mol, respectively, against standard sulphathiazole of −6.2 kcal/mol, having pIC_50_ values of 4.98, 4.47, 4.99, 4.99, and 5.28, and 5.49, respectively (Fig. 13). The pharmacokinetic and QSAR studies revealed that compound **33** was the most potent wound-healing agent.

The healing potential of *Aloe vera* hydrogel enriched with propanediol and triethanolamine was analyzed by Meza-Valle et al. (2021) among 27 female Wistar rats injured with 2 cm back lesions. A hydrogel of hydrophilic and adhesive nature hastened the healing process and reduced healing time by 29% in wounded rats against negative control by boosting fibrinogenesis, angiogenesis and lowering inflammation. Hydrogel-treated rats exhibited rapid wound closure by reducing wounded area by 28.62 ± 6.02% against standard control 18.48 ± 3.12% (after 4 days), 56.52 ± 1.53% against 47.49 ± 3.89% (after 8 days), 91.60 ± 0.99% against 83.99 ± 2.15% (after 15 days), and 100% reduction against 93.58 ± 3.70% (after 21 days), respectively, analyzed via microscopic study.

The beneficial effect of the combination of *Teucrium polium* extract (TPE) and *Aloe vera* gel (AVG) was explored by Gharaboghaz et al. (2020) towards rapid healing of a wound of 7 mm in 108 male BALB/c diabetic rats treated with different doses of TPE (5% and 10%), AVG (5% and 10%), and a combination of TPEO and AVGO (5% + 5%). Administration improved the wound closure by reducing the wound area to 17.3 mm^2^ (5% TPEO), 13.2 mm^2^ (10% TPEO), 18.3 mm^2^ (5% AVGO), 14.1 mm^2^ (10% AVGO), and 10.2 mm^2^ (combo of both) against the reference mupirocin 20.2 mm^2^ at *p* < 0.05. Moreover, the potent dose (5% TPE + 5% AVG) significantly reduced the TNF-*α* levels from 2.8 pg/mL to 1.6 pg/mL and IL-1*β* levels from 10 pg/mL to 5 pg/mL at *p* < 0.05 considerably. The combo dose also statistically enhanced the healing proteins like VEGF, IGF-1, GLUT-1, and FGF-2 and alleviated the MDA levels against the standard control at *p* < 0.05 in 7 days.

Kudłacik-Kramarczyk et al. (2021) reported the composition of *Aloe vera*-modified chitosan-based hydrogel as a promising dressing for wounds in L929 fibroblasts cells. *Aloe vera* juice of high viscosity containing various vitamins, enzymes, saccharides and amino acids etc. used over hydrogel. High-performance hydrogel soaked up more than 15% water, and released 80% of *Aloe vera* (5 h) at pH = 2 because of the enhanced swelling ratio from 1.5 g/g to 1.7 g/g and maximum surface height from 55 µm to > 100 µm. It also improved cell viability and hydrophilicity by decreasing the contact angle from 82.5º to 73º. The composition also acted as an inhibitor of the inflammatory response and was non-toxic.

Chakraborty et al. (2021) evaluated the effect of AVG (extracted by mechanical gel isolation method) enriched with insulin-incorporated nanoemulsion (NE5) on 30 STZ-induced diabetic Wistar rats injured with 8 mm wounds. These rats were given the insulin-based NE5, both with and without *Aloe vera*, for 14 days. Insulin-based NE5 significantly alleviated the blood glucose levels from 314.56 ± 43.00 mg/dL to 203.33 ± 7.33 mg/dL (with *Aloe vera*) and 294.69 ± 15.43 mg/dL to 259.70 ± 34.50 mg/dL (without *Aloe vera*) and elevated the insulin levels from 4.03 ± 0.30 µI/mL to 13.93 ± 0.44 µI/mL (with *Aloe vera*) and 8.56 ± 0.23 µI/mL (without *Aloe vera*) at *p* < 0.0001 over 42 days. In the context of wound healing, the wound closure was improved by 75% (with *Aloe vera*) and 65% (without *Aloe vera*) and exhibited zero irritation, with 90% of skin reforming within 15 days. The innovative approach was found to be stable drug content for almost 6 months ranging from 99.2 ± 1.2% to 97.2 ± 1.4%, having spreadability of 5.92 ± 1.05 cm (with *Aloe vera*) and 5.75 ± 0.96 cm (without *Aloe vera*) and viscosity of 8488 ± 88.31 cP (with *Aloe vera*) and 8571.33 ± 77.67 (without *Aloe vera*), respectively.

Seyrek et al. (2023) reported an alternative approach to diabetic wound healing by using *Aloe vera* drink of 300 mg/kg per day in 21 STZ-induced diabetic rats for 14 days. The blood sugar level was significantly reduced measurably from 367.86 ± 113.55 mg/dL to 220.86 ± 5.49 mg/dL at *p* < 0.05. *Aloe vera* thickened the epidermis layer, enhanced the collagen synthesis and leukocyte infiltration mechanisms, which simultaneously improved the tissue regeneration in the wound area. It also boosted the MMP-1 and TIMP-1 proteins, playing a key role in rapid wound closure.

Naik et al. (2023) formulated and evaluated the wound-healing potential of a bovine serum albumin (BSA) hydrogel modified with *Aloe vera* powder through both *in vivo* and *in vitro* studies. The BSA-*Aloe vera* hydrogel exhibited high porosity, self-fluorescence, non-toxicity, antibacterial and antioxidant properties, promising its therapeutic effects*. *In vitro study, treatment of various concentrations (1, 2, 4, and 8 mg/mL) of hydrogel to 3T3 fibroblast cells revealed zero toxicity; even at higher concentrations, cell viability was 72–76%. The human blood cells were incubated with different concentrations of hydrogel (1 mg/mL and 5 mg/mL) for 2, 4, and 6 h, which demonstrated negligible hemolytic activity of < 5%. In an *in vivo* study, hydrogel was topically administered to 21 STZ-induced female albino rats having dorsal wounds. It significantly accelerated the healing rate to 94.091 ± 1.197% within 21 days by enhancing the concentrations of nitric oxides from 1.085 ± 0.693 µM/mg to 2.433 ± 0.177 µM/mg, SOD enzymes from 0.187 ± 0.094 ng/mg to 1.023 ± 0.015 ng/mg, and GSH 5.008 ± 0.838 nM/mg to 10.199 ± 0.172 nM/mg at *p* < 0.001. Moreover, it also reduced the concentrations of LDH enzymes from 160.133 ± 20.578 nM/mg to 75.771 ± 6.893 nM/mg, *p* < 0.05, GST enzymes from 49.959 ± 5.242 nM/mg to 9.302 ± 1.333 nM/mg, and lesion size from 20.060 ± 0.417 mm^2^ to 1.533 ± 0.310 mm^2^, at *p* < 0.01, respectively.

An alternative perspective of diabetic wound healing by using combined fibroin and *Aloe vera* extracted by lyophilization of its gel was reported by Phimnuan et al. (2023) emphasizing the activation of mitogen-activated protein kinases (MAPKs) such as extracellular signal-regulated kinase (ERK) pathways from diabetic wounded fibroblasts cells. This aloe-modified film significantly enhanced the viability, proliferation, DNA synthase phase, migration, and Vascular Epidermal Growth Factor (VEGF) secretion of diabetic wounded cells from 100% to 215.52 ± 5.22%, 100% to 576.90 ± 6.87%, 40.78 ± 0.50 to 47.92 ± 0.93%, 38.63 ± 2.36% to 93.47 ± 2.86%, and 140.59 ± 62.68 pg/mg to 828.24 ± 87.33 pg/mg, respectively, at *p* < 0.001. It also effectively reduced the cell senescence from 12.43 ± 2.57% to 0.63 ± 0.51% at *p* < 0.001. The mean fluorescence intensity of phospho-p44/42 of MAPK (ERK1/2) was 2082.50 ± 41.41, much higher than the 2% fetal bovine serum intensity of 1611.50 ± 126.74, promising rapid wound healing in diabetic conditions.

Massoud et al. 2022 analyzed the wound-healing potential of an *Aloe vera*-olive oil combination (AVO) among 48 STZ-induced male albino diabetic rats with almost 1 cm^2^ wounds for 14 days. The *Aloe vera* gel was extracted by mechanical isolation method and then mixed with olive oil to make *Aloe vera*-olive oil. The treatment of AVO combination decreased the lesion size from 78.708 ± 4.5% to 0.54 ± 1.1% and the inflammation mediator NF-*κ*B to 9.76% against the standard 16.28% within 14 days. In addition, it also enhanced the cell proliferation and angiogenesis mechanism by elevating the cell proliferation (Ki-67) and angiogenesis (CD34) levels, leading to rapid wound healing.

#### Clinical studies related to diabetic complications

Amin et al. (2024) investigated the anti-inflammatory and wound-healing impacts of *Aloe vera* in managing diabetic foot ulcers through clinical trials including 40 patients for 4 weeks. *Aloe vera* and its combination with Plantago Major significantly reduced the inflammation, promoting angiogenesis and collagen synthesis without any adverse effect.

Sandhiya et al. (2025) assessed a randomized-based clinical trial among 62 T2DM patients (age ranging from 50 to 55 years) having diabetic foot ulcers, treated with 100% w/w of *Aloe vera* gel for 28 days. *Aloe vera* gel obtained commercially, was administered topically on the wound after excisional treatment in comparison with the standard control. *Aloe vera* promises fast and efficient wound healing by reduction of scores from 42 ± 3.11 to 19 ± 2.17 against the standard control from 43 ± 3.58 to 25 ± 2.17 evaluated via the Bates-Jensen Wound Assessment Tool (BWAT) at *p* = 0.0029. In addition, *Aloe vera*-treated group showed better improvement of 86.6% in comparison with the standard control group of 6.6%.

A double-masked random-based clinical trial was performed by Hosseini et al. (2024) to evaluate the analgesic potential of *Aloe vera* gel among 64 patients (of average age 70 years) in decubitus ulcer management for 3 weeks. *Aloe vera* gel dressing applied topically per day significantly reduced the pain score from 5.36 ± 1.13 to 1.00 ± 0.00 against the reference control from 6.65 ± 1.89 to 4.96 ± 0.87 at *p* < 0.001.

**Fig. 14:**
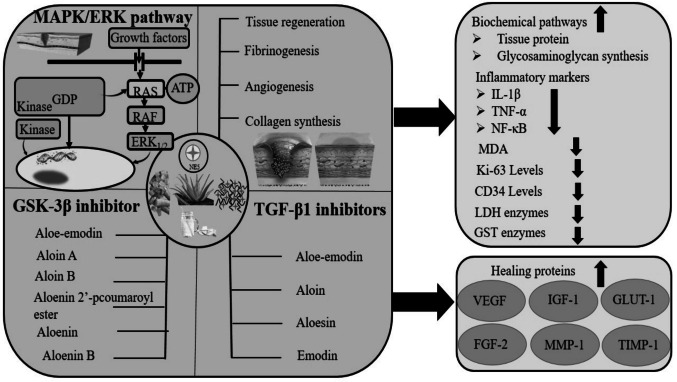
Mechanism of action of *Aloe vera* in wound healing

Wound healing potential of *Aloe vera* explained in Fig. 14 which was followed by enhancing healing rate via tissue regeneration, fibrinogenesis, angiogenesis, collagen synthesis and activating MAPK/ERK1/2 pathways. *Aloe vera* derived active ingredients as reported by Roney et al. (2024a, b) that exhibited the inhibition mechanism of factors GSK-3*β* and TGF-*β*1 also fastened the wound closure and shortened the healing time in diabetic patients. The Table 3 depicts the beneficial impact of *Aloe vera*’s extracts and derived constituents in diabetic complications.

**Table 3:** Summary of beneficial effects of *Aloe vera* in Diabetic complications

Diabetic Complication	Formulation type	Sample size	Dosage & frequency	Duration	Key findings	References
Anti-obesity	*Aloe vera* gel extract	24 male high-fat-diet-treated mice	Orally, 0.2 mg/g per day	10 weeks	Body weight: 41.0 ± 0.5 g to 38.6 ± 0.9 g vWAT: 1,195.5 ± 93.3 mg to 939.3 ± 97.2 mg iWAT: 2,369.6 ± 126.1 mg to 2,196.3 ± 124.8 mg liver weight: 1,675.9 ± 126.1 mg to 1,434.7 ± 107.9 mg	Tada et al. (2020)
	*Aloe vera* leaf extract	40 STZ-induced T2DM male albino rats	Orally, 300 mg/kg	60 days	Body mass: 272.00 g to 249.90 g	Javaid and Waheed (2024)
	*Aloe vera* juice	16 obese men	HF + AVJ	4 weeks	Triglyceride level: 109.5 ± 14.8 mg/dL to 101.7 ± 8.9 mg/dL Fatty acids: 0.76 ± 0.07 mmol/L to 0.74 ± 0.04 mmol/L	Thilavech et al. (2024)
			HF + AVJ + 0.5 g Acemannan		Triglyceride level: 109.5 ± 14.8 mg/dL to 99.3 ± 10.1 mg/dL Fatty acid: 0.76 ± 0.07 mmol/L to 0.61 ± 0.06 mmol/L	
			HF + AVJ + 1 g Acemannan		Triglyceride level: 109.5 ± 14.8 mg/dL to 94.3 ± 9.2 mg/dL Fatty acid: 0.76 ± 0.07 mmol/L to 0.71 ± 0.05 mmol/L	
	*Aloe vera* extract	3T3-L1 cells	10–100 µg/mL	–-	Adipogenesis inhibition: IC_50_ = 43.83 µg/mL; Reduced triglyceride levels and GPDH enzyme at *p* < 0.05. Reduction of gene expression: PPAR*γ*, SREBP-1c, FAS, aP2, C/EBP*α*, HSL, ATGL, ACC levels	Yunusoglu et al. (2022)
Nephroprotective	AV constituent Compound **22**	Human podocytes cells	Not given	–-	IRF4 inhibition: IC_50_ = 8.038 µM Reduced IL-13; IL-7; collagen I levels	Lu and Li (2022)
		18 STZ-induced diabetic sprauge dawley rats	Orally, 20 mg/kg per day	10 weeks	Reduce inflammation, urinary albumin, urinary creatinine, blood urea nitrogen at *p* < 0.01	
	*Aloe vera* butanolic extract	27 STZ-induced T2DM male wistar rats	Orally, 50 mg/kg per day	20 days	Reduced glycemia at *p* < 0.05 within 6 h Regulation of 9 key protein	Santos et al. (2021)
	*Aloe vera* ethanolic extract	21 female STZ-induced diabetic rats	Orally, 300 mg/kg	14 days	MDA: 21.39 ± 8.37 nmol/gm to 12.29 ± 3.74 nmol/gm Reactive oxygen specie: 60.67 ± 10.96 rlu/mg to 54.53 ± 8.12 rlu/mg Bax proteins: 30.50 ± 5.13% to 27.57 ± 3.37% Caspase protein: 32.91 ± 8.79% to 28.24 ± 3.93% Apoptic index: 10.79 ± 3.71% to 6.40 ± 2.27% AQP3 protein: 49.02 ± 12.34% to 57.64 ± 9.22%	Şeker et al. (2023)
	*Aloe vera* ME	30 ethanol-induced male rats	50 and 100 mg/kg	18 days	Creatinine level: 0.41 mg/dL to 0.40 mg/dL (100 mg/kg) Urea level: to 33.52 mg/dL (100 mg/kg) MDA:9.54 mg/dL to 6.70 mg/dL (100 mg/kg) Catalase activity:7.98 mg/dL to 10.43 mg/dL (100 mg/kg) Glutathion activity:5.62 to 7.98 (50 mg/kg)	Dibal et al. (2024)
	*Aloe vera* AE		25 and 50 mg/kg		Creatinine level: 0.41 mg/dL to 0.36 mg/dL; Urea level: to 25.11 mg/dL MDA: 9.54 mg/dL to 7.05 mg/dL Glutathion activity: 5.62 to 7.91 mg/dL (All with 50 mg/kg)	
Neuroprotective	*Aloe vera* + aerobic exercise	32 female diabetic patients (age 40–55 years)	Orally, 400 mg/kg	8 weeks	Blood sugar level: 10.94 ± 0.929 mmol/L to 8.48 ± 0.749 mmol/L Insulin level: 7.46 ± 5.65 mU/mL to 5.77 ± 0.400 mU/mL Insulin resistance: 3.64 ± 0.587 to 2.18 ± 0.340 Body weight: 29.17 ± 1.91 kg/m^2^ to 26.26 ± 2.38 kg/m^2^ BDNF: 160.5 ± 2.81 ng/L to 215.9 ± 9.39 ng/L Insulin sensitivity: 0.525 ± 0.019 to 0.592 ± 0.024 Aerobic capacity: 26.13 ± 1.14 mL/kg/min to 28.27 ± 2.01	Malayeri et al. (2021)
	*Aloe vera* gel	25 STZ-induced male Wistar rats	400 mg/kg per day	8 weeks	Body weight: 205.7 ± 4.2 mg/dl to 248.3 ± 8.6 mg/dl Blood glucose level: 536.7 ± 26.6 mg/dL to 255.4 ± 32.5 mg/dL NGF: 8.48 ± 0.40 to 4.34 ± 0.47 Elevated the TrkA and alleviated the p75 at *p* < 0.05	Mahabady et al. (2021)
	*Aloe vera* ingredients; Barbaloin	30 STZ-induced male diabetic rats	Orally, 25 mg/kg	30 days	Blood sugar level:106.42 ± 3.00 mg/dL to 270.7 ± 6.58 mg/dL Body weight:219.21 ± 3.24 g to 215.8 ± 5.58 g MDA:7.5 nmol/mg to 5.0 nmol/mg; ChAT enzyme:100 U/g to 125 U/g SOD;CAT;GSH: 3.0 U/mg to 4.5 U/mg at *p* < 0.0001 AChE enzyme:7.5 µmolAcSCh/min/mg to 6.0 µmolAcSCh/min/mg Inflammatory mediators: NF-*κ*B: 35 ng/mg to 15 ng/mg; IL-1*β*: 60 pg/kg to 35 pg/kg IL-6: 80 pg/mg to 40 pg/mg; TNF-*α*:150 pg/mL to 75 pg/mL	Omer et al. (2023)
			50 mg/kg		Blood sugar level: 97.25 ± 3.57 to 217.6 ± 2.51 mg/dL Body weight: 219.15 ± 3.36 g to 219.2 ± 5.62 g (50 mg/kg) MDA:7.5 nmol/mg to 3.5 nmol/mg; SOD;CAT;GSH:3.0 U/mg to 5.5 U/mg; ChAT enzyme: 100 U/g to 175 U/g; AChE enzyme: 7.5 to 5.0 µmolAcSCh/min/mg	
Sexual health	*Aloe vera* gel	12 STZ-induced male diabetic wistar rats	380 mg/kg per day	30 days	Blood sugar level: 528.8 ± 27.41 mg/dL to 214.2 ± 14.37 mg/dL Body weight: 195.6 ± 5.98 g to 217.8 ± 17.06 g Testosterone level: 2.32 ± 0.44 ng/dL to 3.19 ± 0.63 ng/dL Sperm counts: 138.2 ± 5.21 n/mm^2^ to 169 ± 6.1 n/mm^2^ Sertoli cells: 1.2 ± 0.2 n/mm^2^ to 1.6 ± 0.2 n/mm^2^ Spermatogonia cells: 78.4 ± 4.2 n/mm^2^ to 84.8 ± 3.51 n/mm^2^ Leyding cells: 7.2 ± 0.58 n/mm^2^ to 7.8 ± 0.37 n/mm^2^ Motile sperm: 3.6 ± 1.12% to 7.4 ± 2.51% Diameter of seminiferous tubule:229.45 ± 6.22 µm to 255.06 ± 6.22 µm and Germinal epithelium layer:16 ± 1.26 µm to 19 ± 1.26 µm	Ghaffari et al. (2023)
		20 BALB/C RATS	10 g and 20 g	10 weeks	Decline the blood glucose level, improved the Leyding cells and spermatogonia cells at *p* < 0.05	Dibal et al. (2023)
	AVHE	32 male diabetic human	1 mg/mL	28 days	Sperm motility:33.03 ± 0.84% to 50.34 ± 0.64% Sperm viability:50.34 ± 0.64%; Chromatin integrity:62.95 ± 0.31% Plasma membrane integrity:36.36 ± 0.95%; Sperm apoptosis: 20.25% SOD activity:23.59%,; Catalase activity:42.87%; TIBARS level: 4.44%	Pal et al. (2024)
			2 mg/mL		Sperm motility: 33.03 ± 0.84% to 50.87 ± 0.43% Sperm viability: 50.87 ± 0.87%,; Chromatin integrity: 62.25 ± 0.23% Plasma membrane integrity: 36.13 ± 0.46%; Sperm apoptosis: 10.72% SOD activity: 23.79%; Catalase activity: 43.48%; TIBARS level:6.26%	
			4 mg/mL		Sperm motility: 33.03 ± 0.84% to 51.33 ± 0.75% Sperm viability:51.33 ± 0.75%; Chromatin integrity: 63.43 ± 0.23% Plasma membrane integrity: 37.60 ± 0.62%; Sperm apoptosis: 9.34% SOD activity:23.92%; Catalase activity:45%; TIBARS level: 6.57%	
		32 male Wistar rats	1 mg/mL		Sperm motility: 24.57 ± 0.42% to 40.72 ± 0.45% Sperm viability: 40.72 ± 0.45%; Chromatin integrity: 61.42 ± 0.41% Plasma membrane integrity: 26.48 ± 0.35%; Sperm apoptosis: 16.02% SOD activity:36.12%; Catalase activity:8.83%; TIBARS level: 7.06%	
			2 mg/mL		Sperm motility:: 24.57 ± 0.42% to 41.23 ± 0.57% Sperm viability: 41.23 ± 0.57%; Chromatin integrity: 61.89 ± 0.89% Plasma membrane integrity: 27.61 ± 0.45%; Sperm apoptosis: 14.37% SOD activity: 39.16%; Catalase activity:9.44%; TIBARS level: 9.44%	
			4 mg/mL		Sperm motility:: 24.57 ± 0.42% to 41.40 ± 0.70% Sperm viability: 41.40 ± 0.70%; Chromatin integrity: 62.23 ± 0.59% Plasma membrane integrity: 28.620 ± 41%; Sperm apoptosis: 7.80% SOD activity: 39.54%; Catalase activity:8.83%; TIBARS level: 8.83%	
Wound healing	*Aloe vera* gel	18 STZ-induced diabetic wistar rats	50 mg/kg/day	10 days	Dry granulation tissue weight:22.5 ± 4.50 mg to 36.5 ± 4.60 mg Wet granulation weight:169.5 ± 10.32 mg to 270.5 ± 12.09 mg Tensile strength:176.36 ± 1.10 g to 317.43 ± 15.07 g Hydroxypyroline tissue:12.18 ± 3.20 mg/g to 14.74 ± 4.62 mg/g Lysyl oxidase enzyme:1129 ± 46 SFU to 1910 ± 61 SFU Tissue protein:25.5 ± 2.60 mg/g to 41.18 ± 4.10 mg/g Hexuronic acid:9.2 ± 1.12 mg/g to 15.21 ± 3.19 mg/g Hexosamin e:7.4 ± 1.40 mg/g to 11.39 ± 2.47 mg/g	Pichaivel et al. (2021)
	BC/AV	15 male sprauge-dawley rats	12.32 ± 3.4% protein of Mol weight 20 kDa	14 days	Protein diffusion:86.765 ± 10.85% (2 h); 97.23% (4 h) Lowered TNF-*α* levels; healing time: 14 days	Yosboonruang et al. (2023)
	AV-derived ingredients	––	––	–-	TGF-* β*1 inhibitor: B.E: −9.6 kcal/mol(Comp **22**); B.E:−9.1 kcal/mol(Comp **13**) B.E:−8.7 kcal/mol(Comp **21**); B.E:−9.6 kcal/mol(Comp **29**)	Roney et al. (2024a)
		––	––	–-	GSK3-*β* inhibitor: B.E:−7.6 kcal/mol(Comp **30**); B.E:−7.8 kcal/mol(Comp **22**) B.E:−8.1 kcal/mol(Comp **31**); B.E:−8.6 kcal/mol(Comp **32**) B.E:−9.5 kcal/mol(Comp **33**); B.E:−8.4 kcal/mol(Comp **34**)	Roney et al. (2024b)
	AV-based hydrogel	27 female wistar rats with 2 cm wound	Hydrogel	21 days	Wound closure: 18.48 ± 3.12% (after 4 days); 47.49 ± 3.89% (after 8 days) 83.99 ± 2.15% (after 15 days); 100% reduction (after 21 days)	Meza-Valle et al. (2021)
			Hyrogel	> 5 h	Soaking of water:: 15% < water and 80% Aloe vera Contact angle: 82.5º to 73º	Kudłacik-Kramarczyk et al. (2021)
		Human blood cells	1 and 5 mg/mL	6 h	Haemolytic activity < 5%	Naik et al. (2023)
		21 STZ-induced female albino rats	Topically administered at wound	21 days	Healing rate:94.091 ± 1.197% Nitric oxide:1.085 ± 0.693 µM/mg to 2.433 ± 0.177 µM/mg SOD enzymes:0.187 ± 0.094 ng/mg to 1.023 ± 0.015 ng/mg GSH:5.008 ± 0.838 nM/mg to 10.199 ± 0.172 nM/mg LDH enzyme:160.133 ± 20.578 nM/mg to 75.771 ± 6.893 nM/mg GST enzyme:49.959 ± 5.242 nM/mg to 9.302 ± 1.333 nM/mg Lesion size:20.060 ± 0.417 mm^2^ to 1.533 ± 0.310 mm^2^	
	Combination therapy	108 male BALB/c diabetic rats with 7 mm wound	TPE (5% + 10%)	7 days	Wound area:17.3 mm^2^ (5% TPEO); 13.2 mm^2^ (10% TPEO)	Gharaboghaz et al. (2020)
			AVG (5% + 10%)		Wound area:18.3 mm^2^ (5% AVGO); 14.1 mm^2^ (10% AVGO)	
			TPEO + AVGO (5% + 5%)		Wound area:10.2 mm^2^ TNF-*α* levels: 2.8 pg/mL to 1.6 pg/mL; IL-1*β* levels: 10 pg/mL to 5 pg/mL	
		Diabetic wounded fibroblasts cells	Fibroin + Aloe vera		Viability:100% to 215.52 ± 5.22%; Proliferation:100% to 576.90 ± 6.87% DNA synthase phase:40.78 ± 0.50 to 47.92 ± 0.93% Migration:38.63 ± 2.36% to 93.47 ± 2.86% VEGF:140.59 ± 62.68 pg/mg to 828.24 ± 87.33 pg/mg Cell senescence:12.43 ± 2.57% to 0.63 ± 0.51%	Phimnuan et al. (2023)
		48 STZ-induced male albino diabetic rats with 1 cm^3^ wound	Aloe vera-olive oil	14 days	Lesion size: 78.708 ± 4.5% to 0.54 ± 1.1% NF-*κ*B: 9.76%	Massoud et al. 2022
	*Aloe vera* drink	21 STZ-induced diabetic rats	300 mg/kg per day	14 days	Blood sugar level:367.86 ± 113.55 mg/dL to 220.86 ± 5.49 mg/dL Boosted the MMP-1 and TIMP-1 protein	Seyrek et al. (2023)
	AVG with insulin-based NE5	30 STZ-induced diabetic wistar rats-8 mm wound	Insulin-based NE5 + Aloe vera	42 days	Blood glucose level:314.56 ± 43.00 mg/dL to 203.33 ± 7.33 mg/dL Insulin level:4.03 ± 0.30 µI/mL to 13.93 ± 0.44 µI/mL Wound closure:75%; Skin reforming:90%	Chakraborty et al. (2021)

The Table 4 explained the clinical trials related to the regulation of metabolic parameters and management of diabetic complication mainly wound healing.

**Table 4:** Summary of clinical studies related to the regulation of metabolic parameters and diabetic complications

Study formulation	Dose	Duration	Population	Key results	References
*Aloe vera* juice	Orally, 20 mL	30 days	60 Indian diabetic patients (T2DM) along with demographic factors	Sugar level: 177.43 ± 17.64 mg/dL to 128.76 ± 27.50 mg/dL (t-value 13.15)	Prakash et al. (2023)
	Orally, 175 mL per day	15 days	12 pre-diabetic patients	Fasting blood glucose: 107.4 mg/dL to 92.1 mg/dL at *p* < 0.001	Budiastutik and Ningsih (2023)
*Aloe vera* drink	Orally, 0.8 g/kg (*Aloe vera*) and 10 g/100 cc (cinnamon)	24 h	60 T2DM	Blood glucose level: 153 mg/dl, 107 mg/dl respectively at *p* < 0.05	Fandizal et al. (2021)
	Orally, 165 g/day	4 weeks	38 participants (age 35–36 years)	Glycated albumin: 19.3 ± 1.73% to 14.9 ± 2.22% HOMA-IR: 3.6 ± 2.04 to 1.9 ± 1.13	Aisya et al. (2022)
*Aloe vera* powder	10 mg	28 days	15 diabetic patients 7 patients (T1DM) 8 patients (T2DM)	Blood glucose level: 261 ± 20.41 mg/dL to 243 ± 7.67 mg/dL (T1DM) 194 ± 7.52 mg/dL to 114 ± 6.96 mg/dL (T2DM)	Younis et al. (2022)
	15 mg			Blood sugar level: 191 ± 8.01 mg/dL to 144 ± 13.27 mg/dL (T1DM) 188 ± 5.95 mg/dL to 135 ± 5.25 mg/dL (T2DM)	
	20 mg			Blood sugar level: 204 ± 8.82 mg/dL to 137 ± 6.82 mg/dL (T1DM) 179 ± 4.76 mg/dL to 161 ± 3.55 mg/dL (T2DM)	
*Aloe vera* gel	100% w/w AVG	28 days	62 T2DM patients (age 50–55 years)	Scores: 42 ± 3.11 to 19 ± 2.17 at *p* = 0.0029 Improvement:: 86.6%	Sandhiya et al. (2025)
	Topically per day	3 weeks	64 patients average 70 years	Pain score: 5.36 ± 1.13 to 1.00 ± 0.00	Hosseini et al. (2024)
*Aloe vera*	*Aloe vera* twice per day	4 weeks	40 patients	Reduced inflammation, enhanced angiogenesis and collagen synthesis	Amin et al. (2024)

## Evidences from meta-analyses and systematic reviews

The studies in the context of glycemic control of the miracle plant, *Aloe vera*, are compellingly supported by multiple meta-analysis reviews, although important distinctions lie in their effects and primary objectives.

Evidence on many medicinal plants was reported by Willcox et al. (2021) who presented many supported clinical trials proving a mean reduction in HbA1c of −0.99% (95% Cl: −1.75, −0.23) with *Aloe vera* leaf gel, placing it at the top of the most efficient natural remedies for long-term glycemic regulation in T2DM. Corresponding to this, the meta-analysis by Budiastutik et al. (2022) demonstrated the statistically significant impact on short-term mediators, determining the alleviation of fasting blood glucose by −1.035 mg/dL in the population. But the focusing point was homogeneity of analysis. Willcox et al. (2021) evaluated the potential effect of *Aloe vera* gel for a long time, but Budiastutik et al. (2022) investigated the statistical heterogeneity of I^2^ = 91.37% in the lowering of FBG, which they associated with variation of study design, especially dosages and time duration almost smaller than 8 weeks. The available meta-analysis suggested that *Aloe vera* had a therapeutic and consistent effect on glycemic control, but short-term FBG was markedly influenced by the variations of prevention protocols (Table 5). Therefore, the prolonged HbA1c reduction is consistently promising, emphasizing the need for further clinical trials to implement standardized preparations, stable and steady doses, and surveillance periods longer than 8 weeks to resolve this time limitation issue.

**Table 5:** Summary of evidences for *Aloe vera* as anti-diabetic medication from meta-analyses and systematic reviews

Study design	Population	Intervention	Primary outcomes	Ref
Overview of Meta-analyses	750 participants (in *Aloe vera* study 235 + 415)	*Aloe vera* gel, juice, powder, given orally with doses range of 600–30000 mg & 200–2800 mg	Mean reduction of HbA1c: −0.99% (95% Cl: −1.75, −0.23) in T2DM	Willcox et al. (2021)
Systematic review and meta-analyses	642 patients	*Aloe vera* capsules with Doses range of 100–1000 mg & 20–300 mL	Lowered fasting blood glucose: SMD: −1.035 mg/dL; 95%; CI: −1.454 to −0.616 High heterogeneity: I^2^ = 91.37% at *p* < 0.001	Budiastutik et al. (2022)
Overview of systematic reviews	1325 participants	*Aloe vera* gel, juice, powder, capsule with Doses range of 15 to 100 mL/day & 300–500 mg twice/day	In T2DM participants: FBG: SMD = −5.61 at *p* < 0.001; HbA1c: MD = −0.95% at *p* = 0.02 In pre-diabetic participants: FBG: SMD = −1.41 at *p* = 0.02; HbA1c: MD = −0.31% at *p* = 0.02 Triglycerides: MD = −4.99 mg/dL; HDL:MD = + 3.66 mg/dL at *p* < 0.001	Araya-Quintanilla et al. (2021)

Araya-Quintanilla et al. (2021) reported the potential of *Aloe vera* as a supplement given orally in various doses ranging from 15 to 100 mL/day or 300 to 500 mg twice daily in the form of gel, powder, capsules, and juice to the 1315 participants having T2DM and pre-diabetes for 4–14 weeks. In T2DM, *Aloe vera* exhibited effective alleviation in fasting blood glucose by SMD as −5.61 at *p* < 0.001 and HbA1c by MD as −0.95% at *p* = 0.02. Moreover, in pre-diabetics, *Aloe vera* was more potent, as it not only demonstrated reduction in fasting blood glucose by SMD = −1.41 at *p* = 0.02 and HbA1c by MD = −0.31% at *p* = 0.02 but also improved the triglycerides by MD = −4.99 mg/dL at *p* < 0.001 and HDL by MD = + 3.66 mg/dL at *p *< 0.001 (Table 5). As a whole, not a single clinical adverse effect was reported, but the short time duration and variable formulation doses limited its applicability at a broader level.

## Negative findings/contradictions of *Aloe vera*

No doubt *Aloe vera* has many health benefits (Kaur and Bains 2024; Catalano et al. 2024), but it also has fewer adverse effects like hepatotoxicity (Delladetsima et al. 2022), nephrotoxicity (Antoine et al. 2023), drug interaction (Mondal et al. 2020), and allergic reaction issues (Matei et al. 2025), calling for caution. Someone should be very careful while using *Aloe vera* at high doses or for long-term use. Nalimu et al. (2021) reported that prolonged use of *Aloe vera* could cause organ toxicity.

### Toxicity

The acute and sub-acute toxicity of the whole leaf of *Aloe vera*, including its extracts, were investigated by Nalimu et al. (2022) among 66 white albino Wistar rats given various doses ranging from 175 mg/kg to 5000 mg/kg for 14 days (acute) and 200 mg/kg to 800 mg/kg per day for 28 days (sub-acute). *Aloe vera* was non-toxic, as no lethal effects or behavior alterations were observed, while LD_50_ was > 5000 mg/kg. However, at high dosages, creatinine was increased from 0.57 mg/kg to 0.83 mg/dL and spleen mass slightly decreased, emphasizing kidney damage. *Aloe vera* was safer for a short time, but long-term usage could cause renal toxicity.

Delladetsima et al. (2022) reported a case of a 59-year-old woman diagnosed with complex hepatic injury over a period of almost 10 years (2005–2015) of daily intake of polyherbal supplements containing vitamin A, fish oils, and *Aloe vera* gel. The patient was accompanied by substantial elevations in ALT level above 227 U/L and AST levels above 153 U/L, but bilirubin and ALP levels were normal. Microscopic study of the liver demonstrated zone 3 necrosis, lipid-based granulomatous inflammation, sinusoidal fibrosis, and stellate cell hyperactivity, indicative of hepatitis. The Roussel Uclaf Causality Assessment Method (RUCAM) score was + 8, exhibiting phytogenic hepatotoxicity probably due to concomitant intake of *Aloe vera* and vitamin A. Cessation of these herb supplements restored liver enzymatic levels within 5 months, revealing the aloe-induced toxicity.

Antoine et al. (2023) evaluated the individual case of a 47-year-old female patient having chronic hepatotoxicity and nephrotoxicity along with acute hepatitis due to taking an *Aloe vera*-derived detox supplement for almost 20 days. Laboratory tests revealed elevation in ALT, ALP, GGT, and total bilirubin by 715 IU/L, 394 IU/L, 500 IU/L, and 21.2 mg/dl, respectively, indicating hepatotoxicity. The RUCAM score was + 8, proving herb-induced liver damage. After 3 weeks, the patient had been induced to nephrotoxicity with hepatotoxicity as worse ALT (156 IU/L), ALP (800 IU/L), GGT (900 IU/L), and bilirubin (62 mg/dL) along with chronic liver diseases, highlighting the adverse effect of *Aloe vera* on the liver and kidney.

### Interaction of *Aloe vera* with standard medication

Mondal et al. (2020) evaluated the interaction of *Aloe vera* with a standard drug by co-administration of *Aloe vera* gel and glimepiride to 153 STZ-induced (45 mg/kg dose) diabetic Wistar rats for 8 days. A high glycemic reduction of 58.80% was observed by the combination of *Aloe vera* and glimepiride (400 mg/kg + 0.144 mg/200 g) given orally for 8 days. In an acute toxicity study, a very high dose of 2000 mg/kg was tolerated, and no death or behavioral alteration was observed within 14 days. However, potential glycemic reduction of the combination could cause hypoglycemia, mandating regular monitoring of glucose and dose adjustment.

## Conclusion

Diabetes mellitus is an alarming and one of the most fatal diseases, characterized by a group of complicated and multicausal pathological physiology. As synthetic drugs had a detrimental effect on vital organs like the kidney, brain, heart, and many more, thus studies shifted toward a new dimension of medicinal plants having fewer adverse effects. In this study, *Aloe vera*, a medicinal plant, exhibited a natural potential as an antidiabetic agent, mainly via enzyme inhibition mechanisms and regulating the biophysiological parameters like blood sugar level, insulin levels, insulin sensitivity, BrdU levels, lipid metabolism, oxidative stress, and inflammatory mediators. In addition, *Aloe vera* and its bioactive compounds proved their potential through a comprehensive series of in silico, in vitro, in vivo, and clinical experiments. Not only glycemic control, but it also showed therapeutic effects towards the mitigation of diabetic-related complications like obesity, nephropathy, neuropathy, and sexual problems. The most emerging issue of chronic diabetic wound healing could also be improved by *Aloe vera*-based hydrogels and dressings involving various mechanistic approaches like anti-inflammatory, antioxidant, tissue formation, and inhibition of GSK3-*β* and TGF-*β*1 pathways. Although several clinical trials indicated that *Aloe vera* significantly affected glycemic control in pre-diabetes and T2DM, but the variability in formulation, doses, trial qualities and duration proved that the evidence was not sufficiently robust in standard manner guidelines. Further research must be aimed at investigating its functional pathways and advanced delivery strategies to understand its comprehensive therapeutic effect. It must emphasize on the standard protocol clinical studies and its bioactive compounds, with safety measurements to evaluate their mechanistic role. Due to its availability and accessibility, *Aloe vera* could prove to be a beneficial complementary treatment for diabetes and its related complications.

## Data Availability

There is no data associated with this article.
